# Characterization of the Genitourinary Microbiome of 1,165 Middle-Aged and Elderly Healthy Individuals

**DOI:** 10.3389/fmicb.2021.673969

**Published:** 2021-08-19

**Authors:** Junjie Qin, Xulian Shi, Junming Xu, Simin Yuan, Bo Zheng, Enpu Zhang, Guixiao Huang, Guo Li, Ganggang Jiang, Shan Gao, Cheng Tian, Ruochun Guo, Zhicong Fu, Qingru Huang, Rentao Yang, Wenyong Zhang, Shenghui Li, Song Wu

**Affiliations:** ^1^Department of Urology, The Third Affiliated Hospital of Shenzhen University (Luohu Hospital Group), Shenzhen, China; ^2^State Key Laboratory of Chemical Oncogenomics, Key Laboratory of Chemical Biology, Tsinghua Shenzhen International Graduate School, Shenzhen, China; ^3^Department of Human Microbiome, Promegene Institute, Shenzhen, China; ^4^School of Medicine, Southern University of Science and Technology, Shenzhen, China; ^5^Teaching Center of Shenzhen Luohu Hospital, Shantou University Medical College, Shantou, China; ^6^Department of Urology and Guangdong Key Laboratory of Urology, The First Affiliated Hospital of Guangzhou Medical University, Guangzhou, China

**Keywords:** genitourinary microbiome, core microbiota, urotypes, co-occurrence network, microbial diversity

## Abstract

Accumulated evidence shows that complex microbial communities resides in the healthy human urinary tract and can change in urological disorders. However, there lacks a comprehensive profiling of the genitourinary microbiota in healthy cohort. Here, we performed 16S rRNA gene sequencing of midstream urine specimens from 1,172 middle-aged and elderly healthy individuals. The core microbiota included 6 dominant genera (mean relative abundance >5%), including *Prevotella*, *Streptococcus*, *Lactobacillus*, *Gardnerella*, *Escherichia-Shigella*, and *Veillonella*, and 131 low-abundance genera (0.01–5%), displaying a distinct microbiome profiles to that of host-matched gut microbiota. The composition and diversity of genitourinary microbiome (GM) were distinct between genders and may fluctuate with ages. Several urotypes were identified by the stratification of microbiome profiles, which were mainly dominated by the six most predominant genera. The prevalence of urotypes was disparate between genders, and the male sample additionally harbored other urotypes dominated by *Acinetobacter*, *Corynebacterium*, *Staphylococcus*, or *Sphingomonas*. *Peptoniphilus*, *Ezakiella*, and *Porphyromonas* were co-occurred and co-abundant, and they may play crucial roles as keystone genera and be associated with increased microbial diversity. Our results delineated the microbial structure and diversity landscape of the GM in healthy middle-aged and elderly adults and provided insights into the influence of gender and age to it.

## Introduction

The “sterile urine” dogma based on traditional cultivation-dependent approaches of bacterial detection has been long questioned. With the development of whole-microbiome next-generation technology, such as 16S rRNA and metagenome sequencing, people get a better understanding of the microbiota composition of urine and its association with health status. Accumulating evidence has shown that microbiota unbalance in urine may be related to lower urinary tract symptoms, such as urinary incontinence (Komesu et al., 2018; Govender et al., 2019), interstitial cystitis (Meriwether et al., 2019), overactive bladder (Wu et al., 2017), and bladder cancer (Bucevic Popovic et al., 2018; Wu et al., 2018), and also other complex diseases like bacterial vaginosis (Gottschick et al., 2017), type 2 diabetes mellitus (Liu et al., 2017a), and prostate cancer (Shrestha et al., 2018).

Apart from the potential roles of genitourinary microbiome (GM) in disease diagnosis, microbial composition of the GM in relatively asymptomatic individuals is an emerging field, with a great value of establishing a benchmark to understand the discriminants of health and disease. Recent research has revealed that GM was dominated by genera like *Lactobacillus*, *Streptococcus*, *Veillonella*, *Staphylococcus*, and *Neisseria* (Aragon et al., 2018; Pohl et al., 2020). The findings were not reproducible among studies because of the limited sample size. Researchers also found that GM composition may differ between genders, age periods, sampling methods used, and microbial niches (Fouts et al., 2012; Lewis et al., 2013; Aragon et al., 2018; Pohl et al., 2020). For example, *Jonquetella*, *Proteiniphilum*, *Saccharofermentans*, and *Parvimonas* genera were only found in the urine of individuals older than 70 (Lewis et al., 2013); male samples tended to be enriched with *Streptococcus*, *Veillonella*, *Staphylococcus*, *Gardnerella*, *Enterobacter* sp., *Neisseria*, and *Haemophilus*, while female samples tended to be abundant of *Lactobacillus* and *Prevotella* (Pohl et al., 2020). Thus, GM research based on a larger samples size and targeted to certain subjects is needed.

Compared with other specimens, such as vagina and gut carrying abundant microbiota, a small number of bacteria (<10^5^ colony-forming units per milliliter) could be found in the urinary tract (Khasriya et al., 2013; Hilt et al., 2014), posing huge challenges in distinguishing the truly existed microbiota signals from possible contaminants. Possible contaminants may arise from commercial biological reagents, cross-sample contamination, sequencing bias, operational pollution, and so on (Karstens et al., 2018). Features, such as being present in negative controls, having the corresponding abundance below a defined threshold, and/or having an inverse correlation with DNA concentration may help uncover contaminants (Karstens et al., 2019). However, there were no standard approaches to mitigate or remove possible contaminants in GM research to date.

In this study, we recruited a large cohort of 1,172 middle-aged and elderly Chinese adults and systematically investigated their GM using 16S rRNA gene sequencing. We assessed the impact of possible contaminants and sequencing depth on microbiome detection. The core microbiota, structure, diversity, and compositional pattern of GM were described in male and female samples, respectively, and their association with different ages was also evaluated.

## Materials and Methods

### Recruitment and Sample Collection

We recruited 1,172 participants between the age of 45 and 86 from Anqing (Anhui Province), Lianyungang (Jiangsu Province), and Wuyuan (Jiangxi Province) from April to December 2016. All participants were asymptomatic of critical diseases, such as cancer, cardiovascular disease (e.g., atherosclerosis, stroke), diabetes, severe obesity (body mass index >30 kg/m^2^), metabolic syndrome, inflammatory bowel disease, or obvious lower urinary tract symptoms. Especially, participants who took antibiotics medication within 30 days or probiotic products within 14 days before sampling were excluded. All participants were required to complete informed consent and questionnaire about their basic information, lifestyle data, and medical history. The study was approved by the institutional review boards of Shenzhen Luohu People’s Hospital. Clean catch midstream urine was collected using a 50-ml urine cup after clean urethral orifice, immediately frozen in dry ice for delivering, then stored at −80°C for subsequent analysis.

### DNA Extraction and Sequencing

About 30–50 ml of midstream urine was used for DNA extraction. After thawing in the lab, the urine sample was centrifuged at 8,000 rpm for 5 min, and the supernatant was discarded. Sterile water was used as negative controls. The DNA extraction was carried out according to the QIAamp DNA Mini Kit protocol, then quantified with Qubit dsDNA HS Assay Kit and stored at −80°C in Tris–EDTA buffer solution. Universal primers, 515F (5′-GTGYCAGCMGCCGCGGTAA-3′), and 806R (5′-GGACTACNVGGGTWTCTAAT-3′), adding with barcode sequences, were used for amplification of V4 regions. PCR mixtures contained 1 μl of each forward and reverse primer (10 μM), 1 μl of template DNA, 4 μl of deoxyribonucleotide triphosphates (dNTPs) (2.5 mM), 5 μl of 10 × EasyPfu Buffer, 1 μl of EasyPfu DNA Polymerase (2.5 U/μl), and 1 μl of double distilled water in a 50-μl reaction volume (EasyPfu DNA Polymerase, Transgen cat. AP211). Thermal cycling consisted of an initial denaturation step at 95°C for 5 min, followed by 30 cycles of denaturation at 94°C for 30 s, annealing at 60°C for 30 s, and extension at 72°C for 40 s, with a final extension step at 72°C for 4 min. Amplicons were run from each sample on an agarose gel. Expected band size for 515F-806R is approximately 300–350 bp. Amplicons were quantified with Qubit dsDNA HS Assay Kit (Thermo Fisher Scientific/Invitrogen, cat. no. Q32854; follow manufacturer’s instructions). The amplicon library for high-throughput sequencing on the Illumina MiniSeq platform was combined in equal amount and subsequently quantified by Qubit dsDNA HS Assay Kit according to the manufacturer’s instructions.

### QC and Removal of Possible Contaminants

Common QC processes included removal of low-quality, artifact and chimeric reads, sequencing error corrections, and read-length trimming. Raw sequencing reads were first removed if they produced >8 homopolymers, >2 mismatches in the primers, or >1 mismatches in the barcode. DADA2 algorithm was performed to remove possible phiX reads and chimeric sequences, correct sequencing errors correction, and generate amplicon sequence variants (ASVs) (Callahan et al., 2016). Then, the remaining reads were truncated from 0 to 150 bases (excluding the primer and barcode sequences) to avoid the sequencing errors at the end. Paired-end reads were connected at the minimum overlap length of 20 bp between the forward and reverse reads, and maximum 2 bp mismatches were allowed inside the overlap zone.

Given that low biomass microbiome may be influenced more by noise of contaminants and cross-talk than high biomass, additional QC processes were required.

(1) Possible contaminants were identified as ASVs with higher prevalence in negative controls than in GUs using decontam package (*p* < 0.5) (Davis et al., 2018).
(2) ASVs were filtered if reads attributed to them in NCs were more than 5% of the total reads for that ASV or if the number of ASVs present in NCs is more than 6% of total samples.
(3) ASVs present in less than three GU samples with low relative abundance (<5‰), which may be introduced randomly by index hopping, were removed.
(4) Only samples with more than 50% reads were kept after filtering.

### 16S rRNA Gene Analysis

Subsequent analysis was based on the quantitative insights into microbial ecology (QIIME2)^1^ platform (Bolyen et al., 2019). Phylogenetic analyses were realized *via* the q2-phylogeny plugin, which performed multiple sequence alignment on the ASV sequences and generated phylogenetic trees of the ASVs from the alignment result. Taxonomic assignment of the ASVs was determined based on a pre-trained Naive Bayes classifier (trained on the Silva; Pruesse et al., 2007; Quast et al., 2013) *via* the q2-feature-classifier plugin. The alpha and beta diversity indexes were calculated based on the ASV composition profiles using the q2-diversity plugin. To avoid sampling depth bias, 4,000 reads were randomly selected from each sample when calculating the ASV and taxa relative abundances.

To compare the microbial composition and function between the urinary and gut microbiota, we downloaded the raw 16S rRNA gene sequencing data of the Guangdong Gut Microbiome Project (GGMP) project (He et al., 2018) from the European Nucleotide Archive database (accession no. PRJEB18535). Data were processed using the same approaches as mentioned above.

### Co-Occurrence Network Analysis

Spearman correlation coefficient between genera was calculated using the *cor* function with “method = spearman” parameter. Enormous interconnection network of the genera with significant correlation (Spearman |rho| > 0.35 and *q*-value < 0.05) was visualized by Cytoscape.

### Functional Profiling

Functional profiling of the genitourinary microbiota was performed using the PICRUSt2 algorithm (Langille et al., 2013). For each sample, the composition of KEGG orthologs (KOs) (Kanehisa et al., 2017) was predicted based on the functional information of the reference ASVs. KEGG module and pathway composition were generated according to the assignment of KOs at https://www.kegg.jp/.

### Statistical Analyses

Statistical analyses were implemented at the R v3.4.2 platform.^2^ Hierarchical clustering was carried out based on relative genera abundance using Bray–Curtis distance and the “ward.D2” algorithm (hclust function in R). The results were assessed for the optimal number of clusters using the Calinski-Harabasz index and mean silhouette width. Spearman correlation coefficient was calculated using the *cor* function with “method = spearman” parameter. Principal component analysis (PCA) was carried out using the ape package in R. Prediction analysis was carried out using the randomForest and pROC packages in R. Chi-square test and Wilcoxon rank-sum test were performed on the R platform, and *p* < 0.05 was considered statistically significant.

## Results

### Study Cohort and Samples Collection

The study cohort included 1,172 Chinese Han adults at ages from 45 to 86 years (mean ± SD, 65.6 ± 8.7 years). All participants were recruited from three regions, including Anqing, Lianyungang, and Wuyuan, located in densely populated areas in east China (Supplementary Figure 1). Of the individuals, 63.7% were female. Participants were excluded if they were symptomatic of serious diseases or urogenital diseases or they took antibiotics or probiotics within 2 weeks before the sampling. Basic characteristics of all participants are summarized in Supplementary Table 1. The midstream voided urine collected contained microorganisms from the whole urinary system and potentially the surrounding genital regions; thus, we used the “genitourinary microbiome” (GM) in the subsequent analysis (Karstens et al., 2018).

### Sequencing Statistics and Possible Contaminants

In order to explore whether there exists abundant microbiota in asymptomatic individuals and its composition, we first examined the concentration of DNA isolated from urine. We found that genitourinary (GUs; mean, 16.28 ng/μl; SD, 11.97) and positive control samples (PCs; mean, 19.09 ng/μl; SD, 8.16) had significantly more DNA mass than negative controls (NCs; mean, 0.13 ng/μl; SD, 0.20, Wilcoxon test *p* < 0.01 in both comparisons), confirming that the genitourinary system is not sterile (Hilt et al., 2014; Supplementary Figure 2).

16S rRNA gene sequencing of 1,172 urine samples generated a total of 47.88 million high-quality sequences (an average of 40,856 sequences per sample; Supplementary Table 1). Totally, we identified 10,230 ASVs existing in all GUs and NCs. Among the 600 ASVs detected in NCs, 472 ASVs were found both in GUs and NCs, and those more prevalent in GUs than in NCs tended to have a relatively higher mean abundance and vice versa (Supplementary Figure 3). A total of 8,100 (79.18%) were seen in at most 3 GU samples, among which 7,900 ASVs were also absent in NCs; thus, these ASVs were likely introduced by index hopping (Hornung et al., 2019).

Given that the signals of low biomass in urine may be influenced by possible contaminants, in addition to conventional steps based on qiime2 plug-ins (Bolyen et al., 2019) (including removing low-quality, artifact, and chimeric reads, correcting sequencing errors, and trimming read-length), we considered multiple factors and performed a more strict strategy to removed possible contaminants that tended to present in NCs or be cross-talk errors (see section “Materials and Methods”). After the multilevel quality-control (QC) processes, 2,475 ASVs remained as real signals, and 7 GU samples producing <50% of average sequencing reads were removed from the following analysis. Totally, a mean of 97.58% reads remained in these 1,165 GU samples, indicating that possible contaminants in our study may pose minor influence to microbiome detection (Supplementary Figure 4).

### General Composition of Genitourinary Microbiome

**FIGURE 1:**
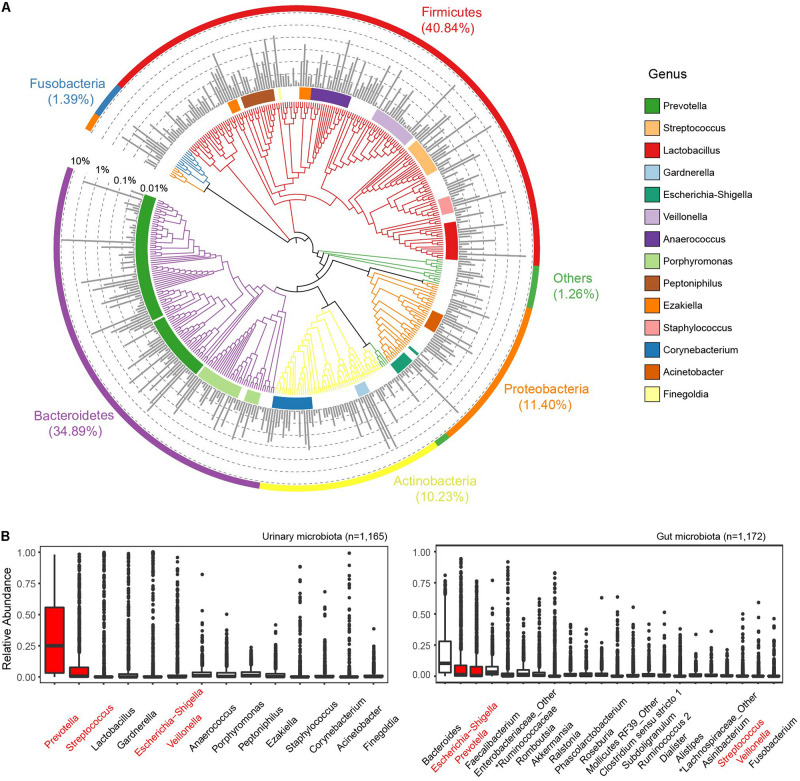
Phylogenetic composition of the genitourinary microbiome. **(A)** The overall landscape of all urine samples from the cohort based on 427 core ASVs. Colored blocks of the circle in nearest phylogenetic tree indicate genera and that of the outermost circle indicated phyla. The middle bar plot shows the average relative abundance of each ASV. **(B)** Comparison of dominant genera (mean relative abundance > 1%) between genitourinary and gut microbiota. The sharing genera are labeled in red.

We identified 427 core ASVs from 1,165 GU samples with mean relative abundance (MRA) larger than 0.01%, accounting for 95.5% of all remaining reads (Figure 1A). ASVs were then taxonomically and functionally annotated using the approaches described in section “Materials and Methods.” At the phylum level, the GM was dominated by *Firmicutes* (MRA, 40.84%), *Bacteroidetes* (34.89%), *Proteobacteria* (11.40%), and *Actinobacteria* (10.23%) (Figure 1A and Supplementary Figure 5). These four phyla constituted 97.3% of all subjects. *Fusobacteria* was the fifth most abundant phylum with MRA of 1.4%. At the genus level, 118 genera with MRA >0.01% (Supplementary Table 2) were identified. These genera represented the dominant GM composition, comprising over 99.29% genera in all samples, suggesting the existence of a “core microbiota” in human urine. Almost the core genera belonged to *Firmicutes* (accounted for 35.59% of genera assigned to this phylum), *Proteobacteria* (21.19%), *Actinobacteria* (19.49%), and *Bacteroidetes* (12.71%), showing a high level of phylogenetic diversity of these phyla in human urine. *Prevotella* (MRA, 0.3122; prevalence, 94.59%), *Streptococcus* (0.1022, 73.91%), *Lactobacillus* (0.0725, 33.65%), *Gardnerella* (0.0650, 43.69%), *Escherichia-Shigella* (0.0634, 45.58%), and *Veillonella* (0.0620, 47.64%) were the most predominant genera in all GU samples (Supplementary Table 3). The richness of the latter five genera was frequently described (Tang, 2017). However, few studies have reported that *Prevotella* was the most dominant and most widely distributed genus in the human urine.

We then compared the GM profiles to the gut microbiome data of 1,172 gender- and age-matched Chinese Han adults from the GGMP cohort (He et al., 2018). Raw sequencing data were downloaded and analyzed using a similar pipeline as the GU samples. A total of 7,789 ASVs were identified in all gut samples, and 4,911 out of these presented in at most 3 gut samples with MRA <0.005 were removed from subsequent analysis. Four hundred sixty-four ASVs were simultaneously observed in the gut microbiome and GUs. Of the 14 dominant genera with MRA >1% in the genitourinary microbiota, only 4 were present among the top 22 most dominant gut genera (Figure 1B). These results highlight the compositional dissimilarity between the urinary and gut microbiotas. However, we identified 1,729 core KOs (cKOs, more than 0.01% ASVs contributing to each cKO) and 316 modules in the genitourinary microbiota (Supplementary Tables 4, 5), and 97.05% cKOs and 96.24% modules of these were shared with the core functions of the gut representing 1,928 cKOs and 320 modules, respectively, demonstrating that the heterogeneous microbiome in these two body sites in asymptomatic individuals may have similar functions.

Except for the aforementioned dominant genera, a large number of genera (*n* = 131 with MRA between 0.01 and 5%; totaling MRA, 34%) resided with relatively low abundances in the urinary tract and likely provided some essential functions in the urine. We revealed a large connection network of the genitourinary microbiota. The low-abundance genera were connected tightly and frequently, but they rarely were linked to the high-abundance genera, indicating crucial roles of the low-abundance microbiota (Supplementary Figure 6).

### Disparity of Genitourinary Microbiome Profiles Between Genders

**FIGURE 2:**
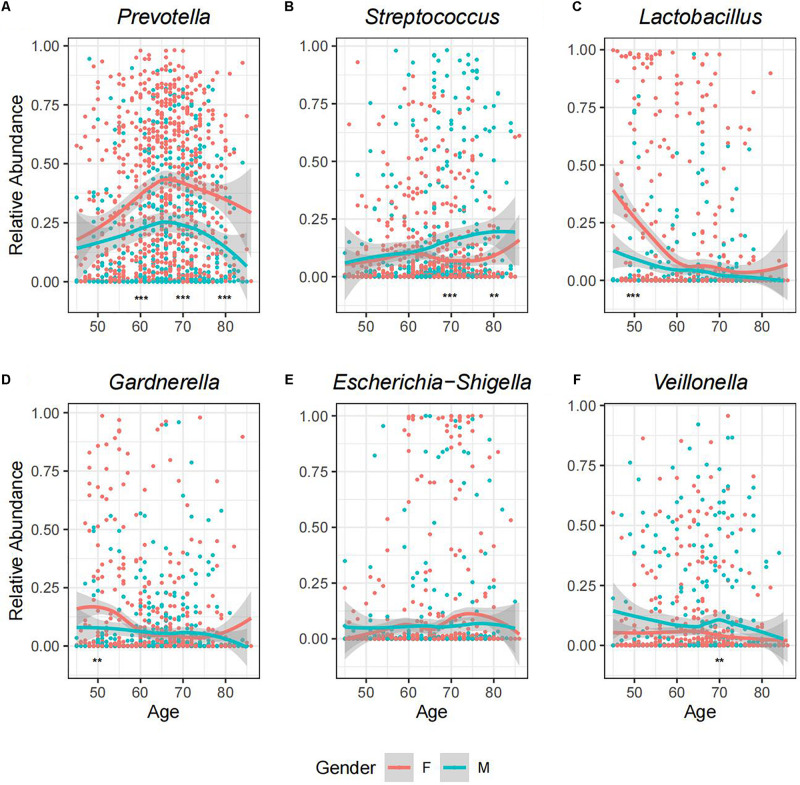
A landscape view of abundance changes in the six most predominant genera with age for both genders. The facets described the MRA of **(A)** *Prevotella*, **(B)** *Streptococcus*, **(C)** *Lactobacillus*, **(D)** *Gardnerella*, **(E)** *Escherichia-Shigella*, and **(F)** *Veillonella*, respectively, along with age for each sample. The smooth curves are formed using the geom_smooth function in R. Relative abundance of each genus was compared between genders at four age stages, and the asterisks indicate the significant level: **p < 0.05 (Wilcoxon test); ***p < 0.01.

A recent research has reported that the microbiome of urine differs between female and male individuals, which could be explained by physiological differences (e.g., anatomical structure, hormone levels, and local defense) (Tang, 2017). Here, we compared the GM profiles between female (*N* = 746) and male individuals (*N* = 419). Among the 14 most dominant genera listed in Figure 1B, we found that MRA of *Prevotella*, *Streptococcus*, *Lactobacillus*, *Veillonella*, *Anaerococcus*, *Ezakiella*, *Staphylococcus*, *Corynebacterium*, and *Acinetobacter* varied significantly between genders (Figure 2 and Supplementary Table 3, Wilcoxon rank-sum test, *p* < 0.05). *Prevotella* was the most dominant genus in both genders but was significantly more prevalent in female samples than in male samples (98.39 vs. 87.83%, Supplementary Table 3, chi-square test *p* < 0.01). Female samples showed relatively higher abundance of *Prevotella*, *Lactobacillus*, and *Anaerococcus*, while male samples exhibited a more abundant proportion of *Streptococcus*, *Veillonella*, *Ezakiella*, *Staphylococcus*, *Corynebacterium*, and *Acinetobacter*. Apart from these dominant genera, the variance between genders was also observed in low abundant genera (Supplementary Table 6). *Eubacterium hallii*, *Senegalimassilia*, *Eubacterium xylanophilum*, *Prevotellaceae Ga6A1*, *Prevotellaceae NK3B31*, and *UBA1819* were seen in at least five female samples but not present in any male samples, while *Pseudoclavibacter*, *Geobacillus*, *Zasmidium cellare*, *Moheibacter*, *Atopobiaceae_Other*, *Deltaproteobacteria_Other*, *Ornithobacterium*, and *Phenylobacterium* existed in at least five male samples but not in any female samples. As we divided the samples into four groups according to their age range, namely, T1 (45–54 years), T2 (55–64 years), T3 (65–74 years), and T4 (≥75 years), we further found that GM disparity between genders may exist in certain age groups (Supplementary Table 3). For example, relative abundance disparities widened markedly at the relatively older age groups for *Prevotella* and *Streptococcus*, while significant variance was observed for *Lactobacillus* and *Gardnerella* in the relatively younger age groups (Figure 2).

**FIGURE 3:**
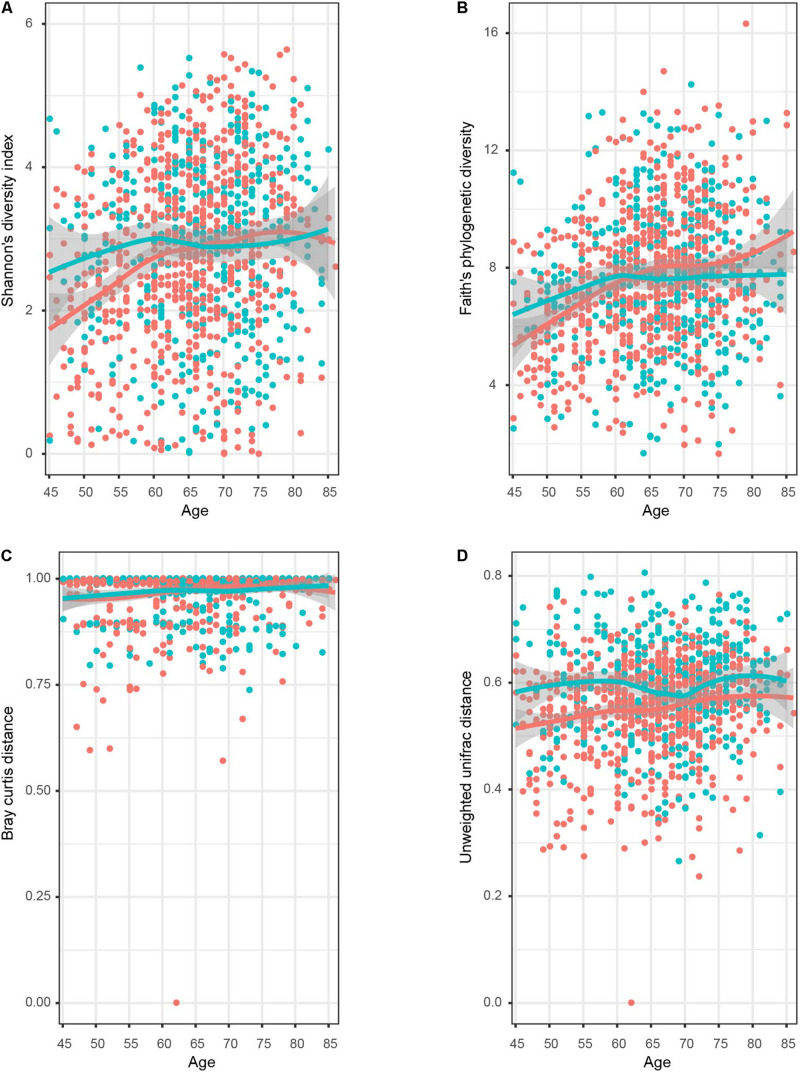
Dynamic response pattern for the microbial alpha and beta diversity of genitourinary microbiome along with the age. The four plots displayed **(A)** Shannon’s diversity index, **(B)** Faith’s phylogenetic diversity, **(C)** Bray–Curtis distance, and **(D)** unweighted UniFrac distance. The smooth curves are formed for female (red colored) and male (blue colored) individuals using the geom_smooth function in R.

To further explore whether the intrasample richness and intersample relationship of genitourinary samples varied between genders, we assessed alpha diversity (including Shannon’s diversity index and Faith’s phylogenetic diversity) and beta diversity (including unweighted UniFrac distance and Bray–Curtis distance) (Figure 3 and Supplementary Figure 7). The Shannon’s diversity index of male samples was significantly higher than the that of female samples (Wilcoxon rank-sum test *p* < 0.05), especially in T1–T2 age groups (Wilcoxon rank-sum test *p* < 0.01 both). On the other hand, all estimators of the beta diversities were significantly higher in male individuals in all age groups compared with the female individuals, suggesting a more diverse genitourinary microbiota in the male population (Supplementary Figure 8).

### Age-Related Genitourinary Microbiome Features

Accumulating evidence has shown that aging and age-related conditions can cause changes in the microbiome composition of urine, which could be explained by changes in nutrition, functional degeneration, diseases, and so on (Liu et al., 2017b; Tang, 2017). To further investigate the impact of age on GM at the bacterial genus level, we compared the microbiome profiles and diversity among samples in different age groups for male and female individuals, respectively (Supplementary Table 3). Generally, in both genders, the composition and proportion of each genus may fluctuate as age changes. It is obvious that the MRA of *Prevotella* increased in T1–T3 groups but flipped in T4 even though its prevalence was comparable; while *Lactobacillus* showed a downward trend with age (Figure 2 and Supplementary Figure 9). For male samples, we found the MRA of *Streptococcus* and *Staphylococcus* climbed, while *Gardnerella* declined with age. T1–T3 and T4 samples showed the highest abundance of *Prevotella* and *Streptococcus*, respectively. Besides, compared to the rest of the cohorts, there were two times more abundant *Lactobacillus* and *Acinetobacter* in T1 and *Staphylococcus* in T4. For the female samples, we observed a significant increase in *Porphyromonas*, *Peptoniphilus*, and *Ezakiella* and a decrease in *Lactobacillus* with age. Compared to other age cohorts showing the highest abundance of *Prevotella*, T1 showed the most enrichment of *Lactobacillus*, which was about three times more stages than other cohorts. The sharp fall of *Lactobacillus* in T2–T4 women was consistent with a previous study (Liu et al., 2017b) and may resulted from the hormonal changes after menopause. T1 group was also two times more enriched with *Gardnerella* than any other three stages in the female samples, but the variance was not seen in the male samples.

No dramatic difference in Shannon’s diversity was observed among male samples in different age groups (Figure 3), but the male T1 cohort showed relatively lower diversity compared to others in terms of Faith’s phylogenetic diversity (Supplementary Figure 7). On the contrary, it is obvious that both two estimators of alpha diversity fortified significantly as age increased in the female samples, as shown in Figure 3 and Supplementary Figure 8. Older groups had significantly higher beta diversity than younger groups for both genders.

### Urotypes Decomposed From Genitourinary Microbiome

**FIGURE 4:**
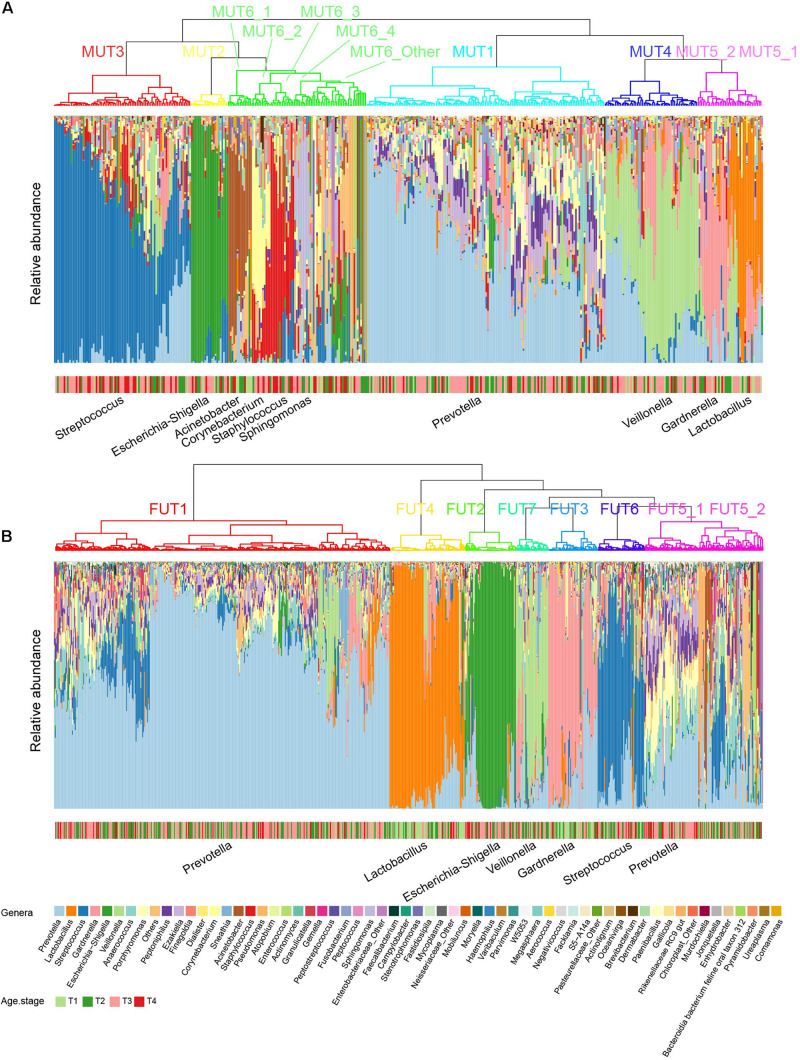
Urotypes of genitourinary microbiome for **(A)** male and **(B)** female samples, respectively. Clustering of the samples was performed based on the Bray–Cutis distance using the “ward.D2” clustering algorithm in R. For individual genitourinary microbiota, the relative abundance of 60 most abundant genera (mean relative abundance > 0.1%) is shown, and all others are summarized as “Others.”

**FIGURE 5:**
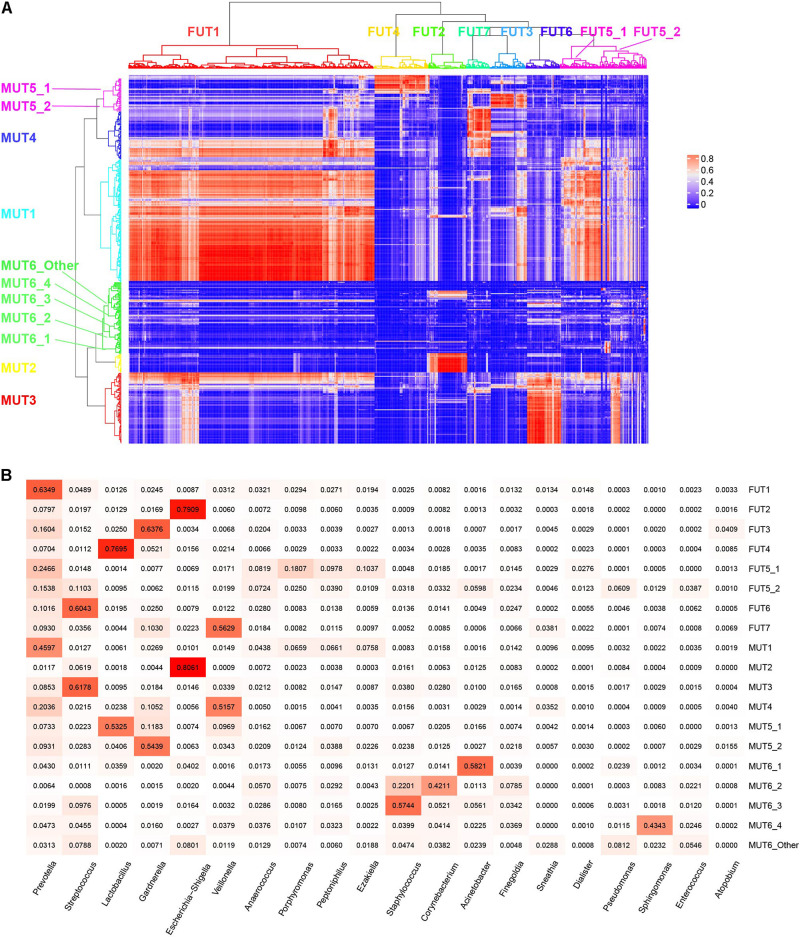
Characteristics of each urotype. **(A)** Correlation of relative abundance between female and male urotypes. **(B)** Heatmap showing the mean relative abundance of representative genera for each urotype.

The term “urotype” was first introduced in 2014 to describe the clusters with certain features of combined microbiota profiles (Pearce et al., 2014; Gottschick et al., 2017). We identified six and seven clusters in male and female samples, respectively, based on hierarchical clustering of the Bray–Curtis distance of rich genera’s relative abundance patterns (MRA > 0.1% for each genus included; others were combined as “Others” group), some of which could be further grouped into subclusters (Figure 4). The appropriate number of clusters was decided by Calinski-Harabasz index and mean silhouette width analyses (Supplementary Figures 10, 11). We identified urotypes in both genders that were severally dominated by *Streptococcus* (MUT3, FUT6), *Escherichia-Shigella* (MUT2, FUT2), *Lactobacillus* (MUT5_1, FUT4), *Veillonella* (MUT4, FUT7), *Gardnerella* (MUT5_2, FUT3), *Prevotella* (MUT1, FUT1, and FUT5_1), and mixed profiles (MUT6_Other, FUT5_2) (Figure 5B and Supplementary Figure 12), which have been noticed in the previous studies (Thomas-White et al., 2016; Gottschick et al., 2017; Aragon et al., 2018). The *Prevotella*-dominant clusters also displayed remarkable abundance of co-occurred *Peptoniphilus*, *Ezakiella*, and *Porphyromonas*. The male samples additionally harbored other urotypes dominated by *Acinetobacter*, *Corynebacterium*, *Staphylococcus*, or *Sphingomonas* (MUT6_1-4). The shared urotypes between two genders had high similarity to each other (Figure 5A). *Prevotella*/*Peptoniphilus*/*Ezakiella*/*Porphyromonas*-dominant urotype was commonest and much more frequent in the female samples than in the male samples (54.96 vs. 33.65%, chi-square test *p* < 0.01). *Lactobacillus*-dominant urotypes accounted for 10.59 and 5.01% in the female and the male samples, respectively, while *Streptococcus* and *Veillonella*-dominant urotypes were more frequent in the male samples with the proportions of 19.33 and 13.13%, respectively. The prevalence and disparity of these major urotypes between genders were concordant with their representative genus. *Acinetobacter*- and *Staphylococcus*-dominated urotypes were enriched within T1–T2 and T3–T4 male samples, respectively, while *Gardnerella*- and *Lactobacillus*-dominated urotypes showed high prevalence in the T1–T2 samples, also agreeing with the association between genera and age groups (Supplementary Figure 13).

We investigated the distribution model of the dominant genera to understand how the urotypes were formed. The abundance of *Prevotella* was close to log-normal distribution (Supplementary Figures 14, 15), which was likely formed by a single process. However, the abundance distributions of the other five dominant genera were bimodal or multimodal, and their high-abundance modals were concentrated in samples from corresponding urotypes, suggesting that the abundances of these genera were generated by two or more distinct processes, one of which likely playing a dominant role in the urotypes formation while the other contributing to the less abundant genera. *Prevotella/Peptoniphilus*/*Ezakiella*/*Porphyromonas*-dominant urotype displayed the highest alpha diversity compared to other urotypes (Supplementary Figure 16), demonstrating the existence of diverse low-abundance genera. *Escherichia-Shigella*-dominant urotype showed the lowest alpha diversity, while *Gardnerella*- and *Veillonella*-dominant urotype showed the lowest beta diversity (Supplementary Figure 16).

### Co-Occurrence and Co-Abundance of *Peptoniphilus*, *Ezakiella*, and *Porphyromonas* as Keystone Genera Promoted Genitourinary Diversity

**FIGURE 6:**
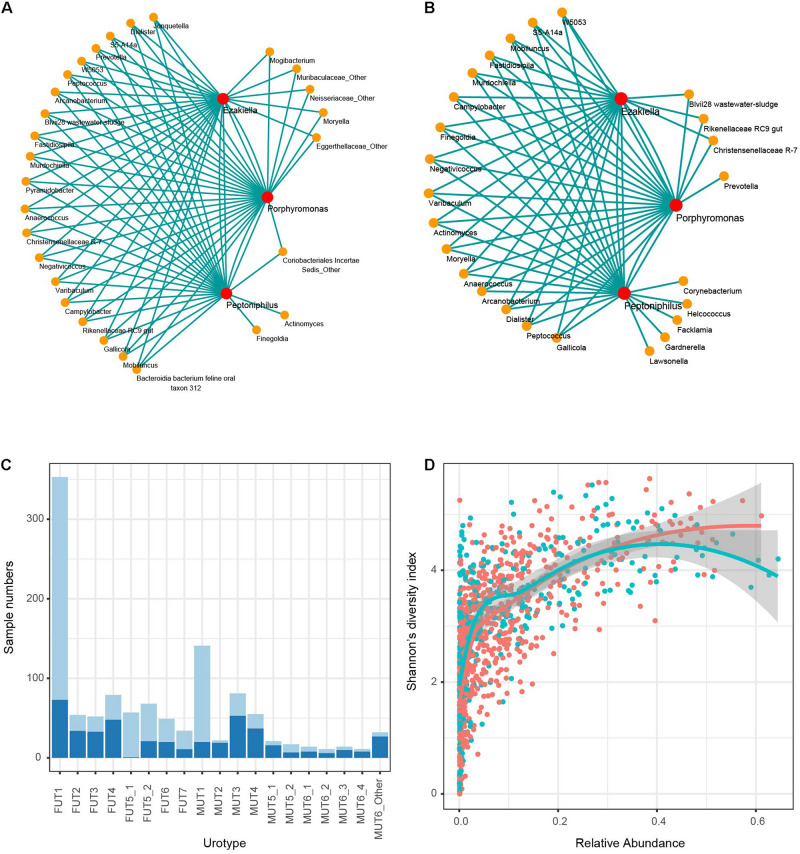
Co-occurrence of *Peptoniphilus*, *Ezakiella*, and *Porphyromonas*. Network of co-occurring genera with *Peptoniphilus*, *Ezakiella*, and *Porphyromonas* based on Spearman correlation analysis for **(A)** male and **(B)** female samples, respectively. Lines connected two genera with Spearman |rho| > 0.35. **(C)** The proportion of samples harboring the co-occurred pattern in each urotype (light blue). **(D)** Correlation distribution of Shannon’s diversity index and relative abundance of the three co-occurred genera. Female and male samples were marked red and blue, respectively.

The co-occurrence patterns of *Peptoniphilus*, *Ezakiella*, and *Porphyromonas* were observed in 505 female and 208 male samples, respectively (chi-square test *p* < 0.01), and these three genera were co-abundant with strong positive correlation higher than 0.75 (Supplementary Figure 17). Co-occurrence network analysis further showed that *Peptoniphilus*, *Ezakiella*, and *Porphyromonas* highly connected with other low-abundance genera, demonstrating their roles as keystone genera (Figures 6A,B). The co-occurrence and co-abundance patterns were typical of MUT1, FUT1, and FUT5_1, accounting for 85.82, 79.32, and 98.25% of corresponding samples (Figure 6C). We further found that the co-abundance of these three genera was positively associated with Shannon’s diversity index, which was used to measure microbial richness (Pearson’s correlation coefficient 0.60 and 0.65 for male and female samples, *p* < 0.01 in both), which could explain the higher diversity of MUT1, FUT1, and FUT5_1 (Figure 6D).

Additionally, we found several keystone genera in the genitourinary samples, including *Dorea*, *Rikenellaceae RC9 gut*, *Ruminococcaceae UCG-002*, *Mobiluncus*, *Campylobacter*, *W5053, S5-A14a*, *Peptococcus*, and *Ruminococcus 1* (Supplementary Figure 18). Different from *Peptoniphilus*, *Ezakiella*, and *Porphyromonas*, these keystone genera were low abundant and contributed minimally to genitourinary microbiota diversity.

## Discussion

In the present study, we applied 16S rRNA gene sequencing of clean-catch midstream urine samples from a large cohort of Chinese between 46 and 85 years to profile the GM of asymptomatic individuals, including 419 male and 746 female samples after filtering. The cohort size was unprecedented especially for male samples, making it possible to detect smaller communities with significance and describe a more comprehensive atlas of GM and relation between microbes and phenotypes, such as gender and age.

To assess the impact of different types of possible contaminants while eliminating the effects of sequencing depth and sample size, we classified all samples into seven groups according to raw sequencing depth (G1-7: ≤20,000, 20,000–30,000, 30,000–40,000, 40,000–50,000, 50,000–60,000, 60,000–70,000, 70,000–80,000, ≥8,000), and extracted 110 samples from each group followed by the same contaminant identification operations. We found comparable reads proportion accounting for distinct contaminants and that lower sequencing depth may bring about low-abundant ASVs in real samples that existed in NCs, while higher depth introduced more frequent chimeras (Supplementary Figure 19). Low sequencing depth <20,000 (G1) may cause relatively higher positive false ASV calls, which were not supported by other groups. It is obvious that higher sequencing depth and larger sample cohort mean stronger detection power in a more comprehensive GM atlas, and how to balance cost and detection sensitivity have been raising accumulative concerns. Further comparison of detection sensitivity demonstrated that samples with more than 40,000 reads (≥G4) may show remarkable detection sensitivity, and the sample size larger than 70 could reach 90% sensitivity of ASVs detection in 100 samples (Supplementary Figure 20). In conclusion, we recommend at least 40,000 sequencing reads and a study cohort size larger than 70 in an attempt to draw a more comprehensive view of GM atlas or other specimens in the low biomass field.

The predominant genitourinary phyla (*Firmicutes*, *Bacteroidetes*, *Proteobacteria*, *Actinobacteria*, and *Fusobacteria*) were the most abundant members in multiple body sites as revealed by the Human Microbiome Project (Human Microbiome Project Consortium, 2012; Lloyd-Price et al., 2017), comprising >95% of overall community configurations. Especially, *Firmicutes* and *Bacteroidetes* contributed approximately 74% of relative abundance in genitourinary microbiota, which was similar compared with the gut microbiota (consisting of ∼90% *Bacteroidetes* and *Firmicutes*) but quite different from the oral cavity (consisted mostly with *Firmicutes*, *Actinobacteria*, and *Proteobacteria* but rarely with *Bacteroidetes*) or vagina (>95% *Firmicutes*) microbiotas. On the genus level, however, the genitourinary microbiota was quite different from the gut microbiota in gender- and age-matched individuals (He et al., 2018). These results highlight the dissimilarities of core microbiota between the urinary tract and other body sites, which was probably determined by the difference in ecological habitats.

Six genera, including *Prevotella*, *Streptococcus*, *Lactobacillus*, *Gardnerella*, *Escherichia-Shigella*, and *Veillonella*, were the most abundant in our cohort. *Lactobacillus*, *Gardnerella*, and *Streptococcus* were generally recognized as the primary urinary residents in the previous studies (Whiteside et al., 2015; Tang, 2017; Aragon et al., 2018). The mean relative yield of *Prevotella* reached 31.22% in our cohort, but rare observation of the most abundant *Prevotella* in urine has been previously reported. Liu et al. (2017b) performed 16S rRNA sequencing targeting V3−4 hypervariable region of urine samples from type 2 diabetes and asymptomatic Chinese women, and found that *Prevotella* was the most predominated genus reaching MRA of ∼15% regardless of age and disease. These findings indicated that enrichment of *Prevotella* in urine may be typical in the Chinese population, and the disparity of its MRA may be caused by sample size, sample collection approaches, amplicon region selection, diet habitat, age, and so on. *Prevotella* is a common type of commensals in several other body sites, such as the oral cavity, gut, and vagina, and an enterotype of gut microbiome was featured by a high proportion of *Prevotella* (Arumugam et al., 2011; Wolfe et al., 2012). *Prevotella* has been associated with lower risk of developing chronic kidney disease (Jiang et al., 2017) and kidney stone (Kelsey, 2016), which agreed with the phenomena of abundant *Prevotella* in asymptomatic samples to a certain degree and indicated its crucial roles in balancing flora in the genitourinary system.

It has been long recognized that microbiome of urine differs between female and male individuals due to physiological differences (Tang, 2017). We found that female samples tended to be more enriched of *Prevotella* and *Lactobacillus*, while male samples exhibited a more abundant proportion of *Streptococcus*, *Veillonella*, *Ezakiella*, *Staphylococcus*, *Corynebacterium*, and *Acinetobacter*, some of which agreed with previous findings (Tang, 2017). Around half of female samples were dominated by *Prevotella*, and 10% of that were dominated by *Lactobacillus*, whereas ∼34, ∼20, and ∼13% of male samples displayed high abundant *Prevotella*, *Streptococcus*, and *Veillonella*, respectively. *Lactobacillus* is a dominant member of the female reproductive tract comprising a relative abundance of over 97% in the lower reproductive tract samples (e.g., lower vagina, posterior fornix, and cervical mucus) (Koedooder et al., 2019), partly explaining its higher abundance in female individuals’ midstream urine samples that could be introduced from women’s vagina. On the other hand, the male samples generally showed a relatively more diverse genitourinary microbiota pattern than female samples. A total of 10 urotypes were identified in our cohort, 6 of which were featured by the dominance of the aforementioned most dominant genera for each and were shared between genders. Urotypes dominated by *Acinetobacter*, *Corynebacterium*, *Staphylococcus*, or *Sphingomonas* were found in healthy men only and accounted for 11.93% of male samples, which could explain the higher diversity of male than female samples to a certain degree. The prevalence of each urotype and the association between urotypes and age groups were highly influenced by its representative dominant genus. The classification of the human GM into distinct urotypes provides an attractive framework for understanding microbial variation in health and disease.

Apart from gender, it is also widely recognized that aging and age-related conditions could affect GM composition. A few studies reported that the proportion of *Lactobacillus* in female individuals’ urine decreased after menopause due to hormone change (Whiteside et al., 2015; Tang, 2017). Consistent with the previous findings, we observed a threefold decrease in *Lactobacillus* after 55 years old (about the age of natural menopause). The decrease in *Lactobacillus* after 55 years old was also seen in male samples, the implementation of which is unknown and awaits more studies. Additionally, we noticed a significant decline in the relative abundance of *Acinetobacter* and *Gardnerella* after 55 years old in several male and female samples. *Gardnerella* is an anaerobic bacterium residing in the normal vaginal flora (Hartmann, 1987), and like *Lactobacillus*, its change may be also influenced by host hormone level. *Streptococcus* was noted to have a sharp increase among male samples over 75 years of age. Generally, the microbial diversity increased with age irrespective of gender in our study. However, the underlying mechanism of genitourinary microbial changes with age is still poorly understood and needs more systematic research with multiomic data. As certain age period may have its own representative genera profile, we further attempted to predict age using GM profiles *via* randomForest method. After extracting 80% samples as the training dataset and used the rest as the test dataset, we predicted age and compared them with corresponding true values for both genders, respectively (Supplementary Figure 21). The correlation between true and predicted age was 0.19 and 0.33 for males and females samples, respectively (Supplementary Figure 22). The relatively low correlation may be explained by the relatively narrow age range in our study population (middle-aged to the elderly), and the GM profile may be promising to separate elderly samples from much younger samples.

Of note, we found the co-occurrence and co-abundance of *Peptoniphilus*, *Ezakiella*, and *Porphyromonas* in 713/1,165 genitourinary samples. These three genera were network hubs that highly connected with each other and with other genera, demonstrating their roles in maintaining the function of the microbial community as keystone genera. We also found that the co-abundance pattern was positively associated with Shannon’s diversity index, emphasizing their crucial roles in promoting diversity.

The major limitation of this study was the bare phenotype information that could help illuminate the underlying mechanism. We have been attempting to collect healthcare records data, conduct questionnaires, and obtain other omic data to make the function of microbiota in urine and the interaction between the GM and host clearer.

In summary, we applied a multilevel pipeline to remove possible contaminants and delineated a landscape of the genitourinary microbiota in a large cohort of healthy middle-aged and elderly adults. Our work could provide a reference for data processing in the field of low-biomass microbiome. The results of this study should greatly expand what is known about urinary microbial diversity and variation and offers new insight into the GM in light of gender and age.

## Data Availability Statement

The datasets presented in this study can be found in online repositories. The names of the repository/repositories and accession number(s) can be found below: https://www.ncbi.nlm.nih.gov/, PRJEB36679.

## Ethics Statement

The studies involving human participants were reviewed and approved by the institutional review boards of the Shenzhen Luohu People’s Hospital. The patients/participants provided their written informed consent to participate in this study.

## Author Contributions

JQ and SW: conceptualization, project administration, and supervision. SY, EZ, GH, GL, GJ, and SG: clinical diagnoses and sample collection. ZF, QH, and RY: experiments. XS and SL: data analysis and writing. XS, JX, BZ, CT, and RG: data analysis and statistical analysis. WZ: editing. All authors read and approved the final manuscript.

## Conflict of Interest

The authors declare that the research was conducted in the absence of any commercial or financial relationships that could be construed as a potential conflict of interest.

## Publisher’s Note

All claims expressed in this article are solely those of the authors and do not necessarily represent those of their affiliated organizations, or those of the publisher, the editors and the reviewers. Any product that may be evaluated in this article, or claim that may be made by its manufacturer, is not guaranteed or endorsed by the publisher.

## Footnotes

1
https://qiime2.org/

2
https://www.r-project.org/
