# Supplementary material for: Characterization of the Genitourinary Microbiome of 1,165 Middle-Aged and Elderly Healthy Individuals
Source: Front Microbiol. 2021 Aug 19;12:673969. doi: 10.3389/fmicb.2021.673969 (PMC8417382; doi:10.3389/fmicb.2021.673969)
Supplement: Supplementary file 1 [file Presentation_1.pdf]

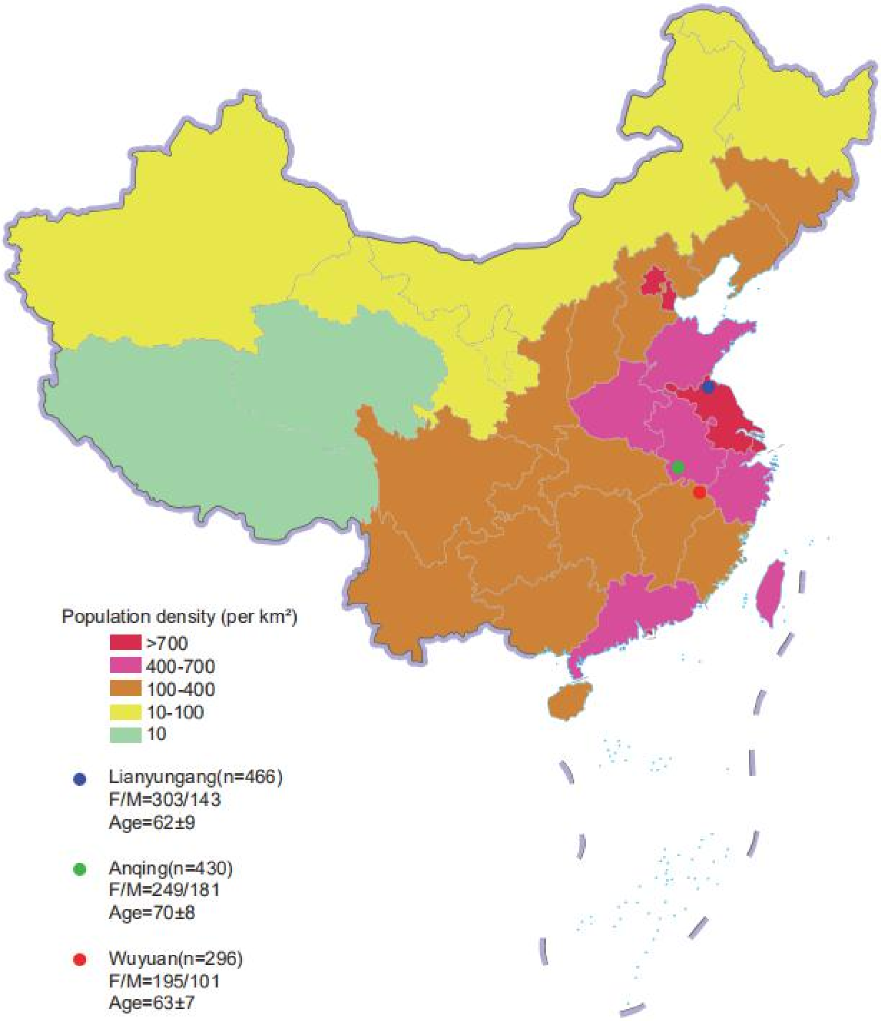

Figure S1. Geographical distribution of the three sampling sites in this study. Participants were recruited from three regions, including Anqing, Lianyungang and Wuyuan, located in densely populated areas in east China.

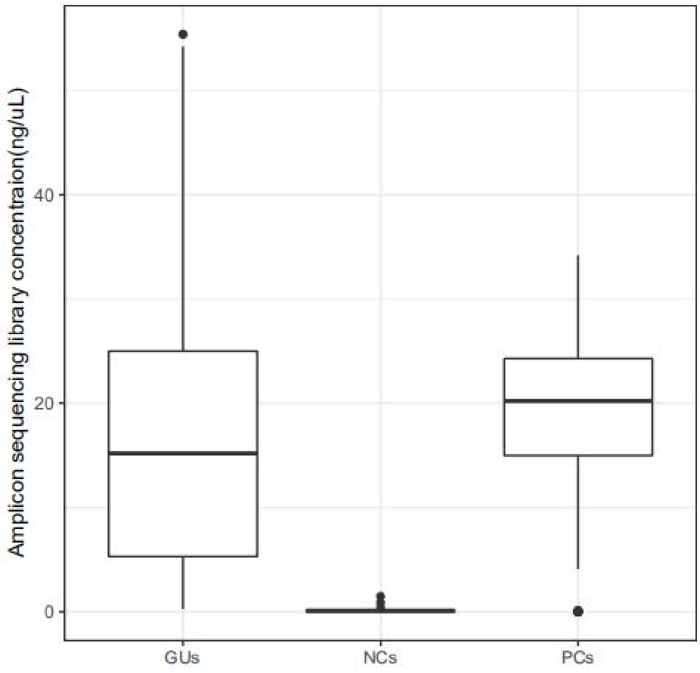

Figure S2. A comparison of concentation of amplicon sequencing library between genitourinary samples (GUs), Negative controls (NCs) and Positive controls (PCs).

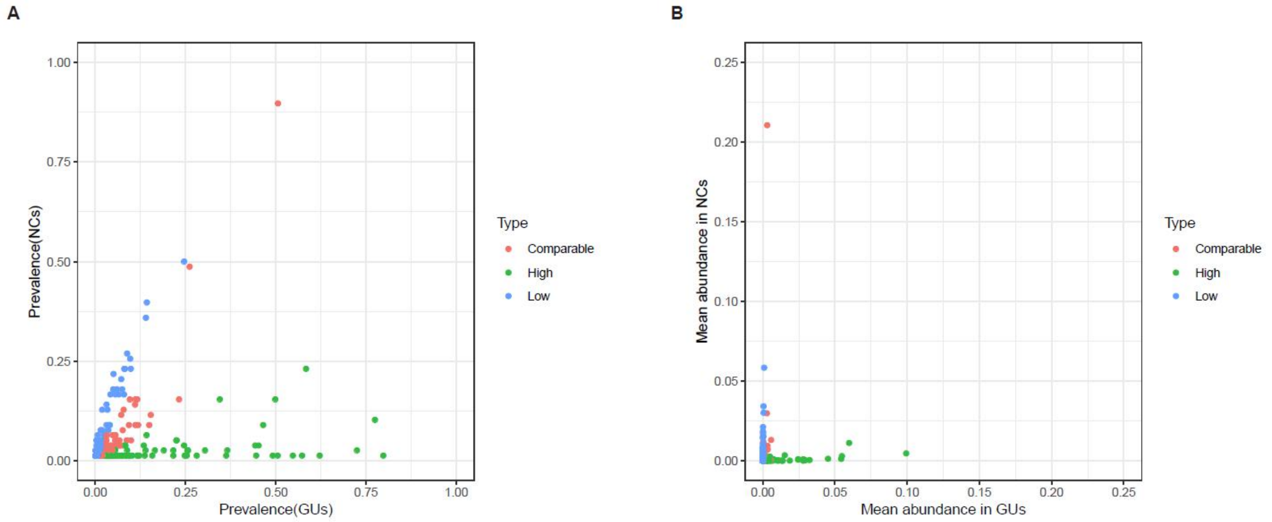

Figure S3. Prevalence (A) and mean relative abundance (B) of ASVs existed both in genitourinary samples and negative controls. These ASVs were classified into 3 groups according to the ratio of prevalence in GUs to that in NCs and were showed in different colours respectively. Green, ratio larger than 2; Blue, ratio smaller than 0.5; Red, ratio between 0.5 to 2.

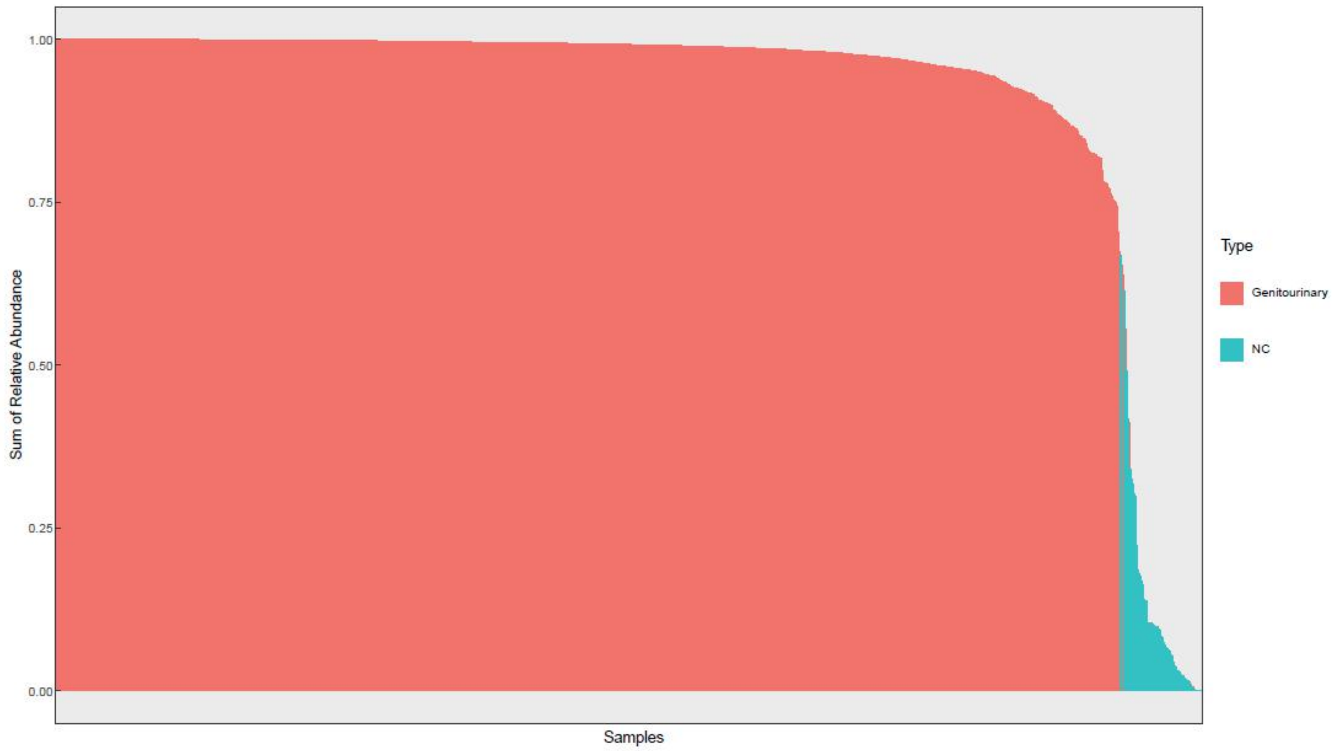

Figure S4. The ratio of reads after filtering to those before filtering for each sample. 7 samples with ratio less than 0.5 were filtered.

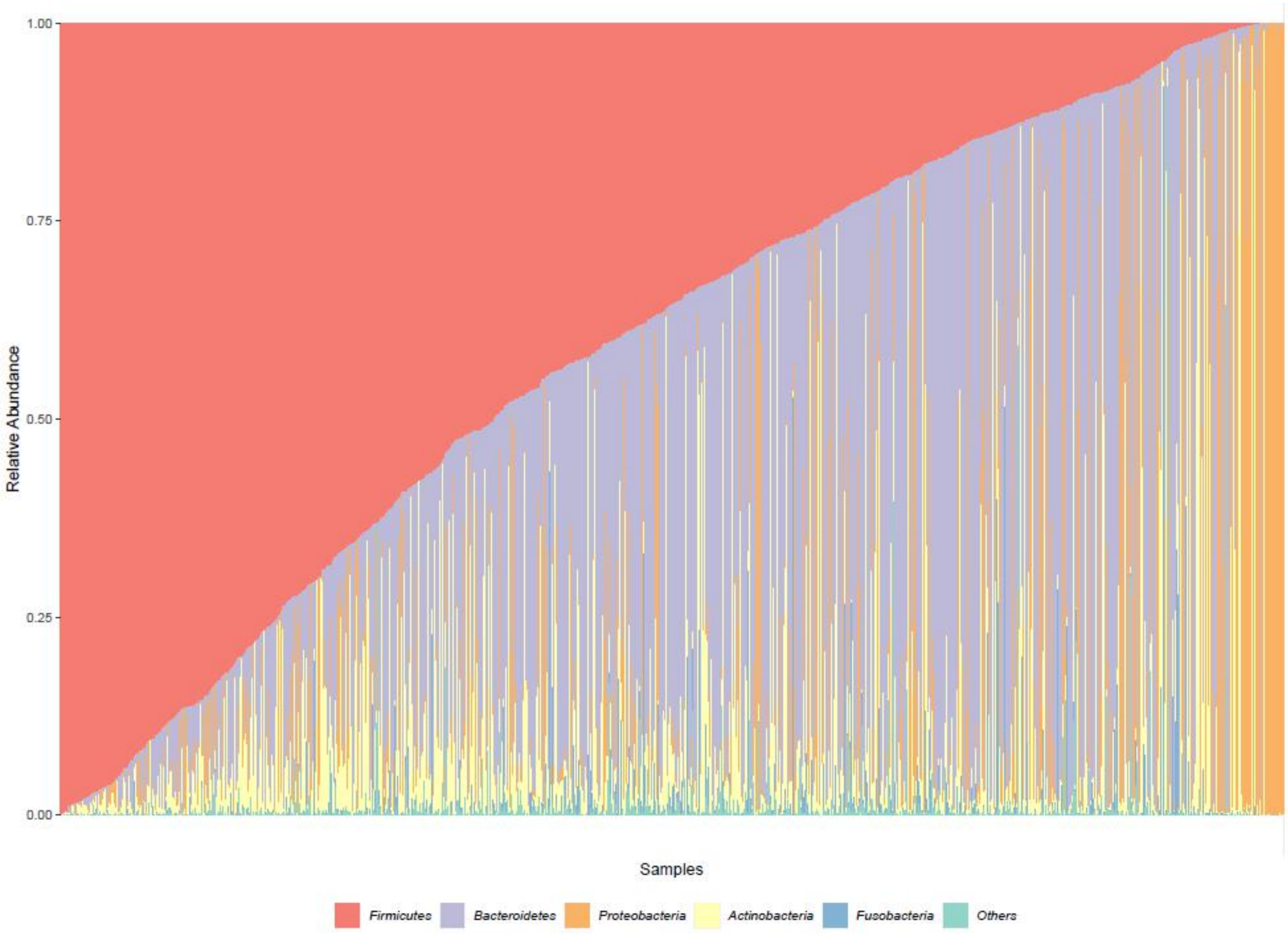

Figure S5. Composition of the genitourinary microbiome at the phylum level.

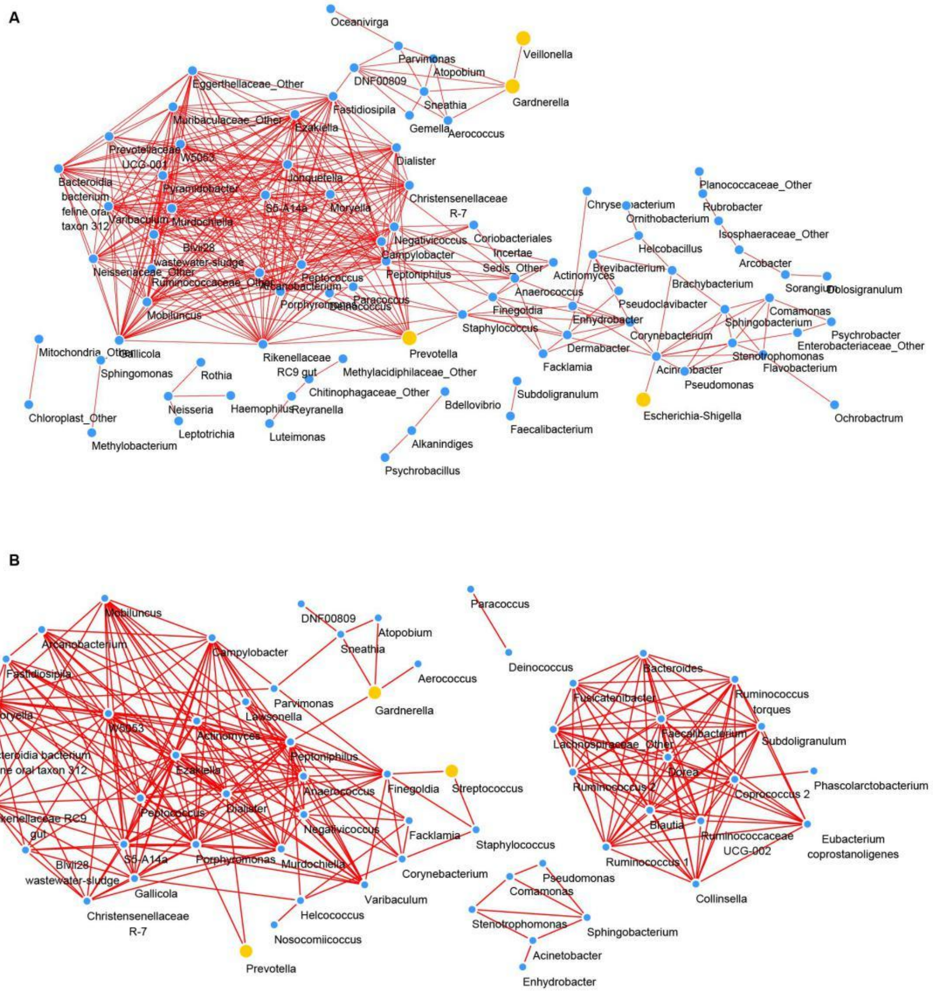

Figure S6. Interconnection of the low-abundance genitourinary microbiota for male (A) and female (B) samples respectively. Enormous interconnection network among all genera (mean relative abundance > 0.01%) of the urinary microbiota. Colored nodes indicate genera; and the low-abundance and dominated genera are labeled in blue and yellow, respectively. Lines connected two genera with significant correlation (Spearman |rho| > 0.35 and p-value < 0.05).

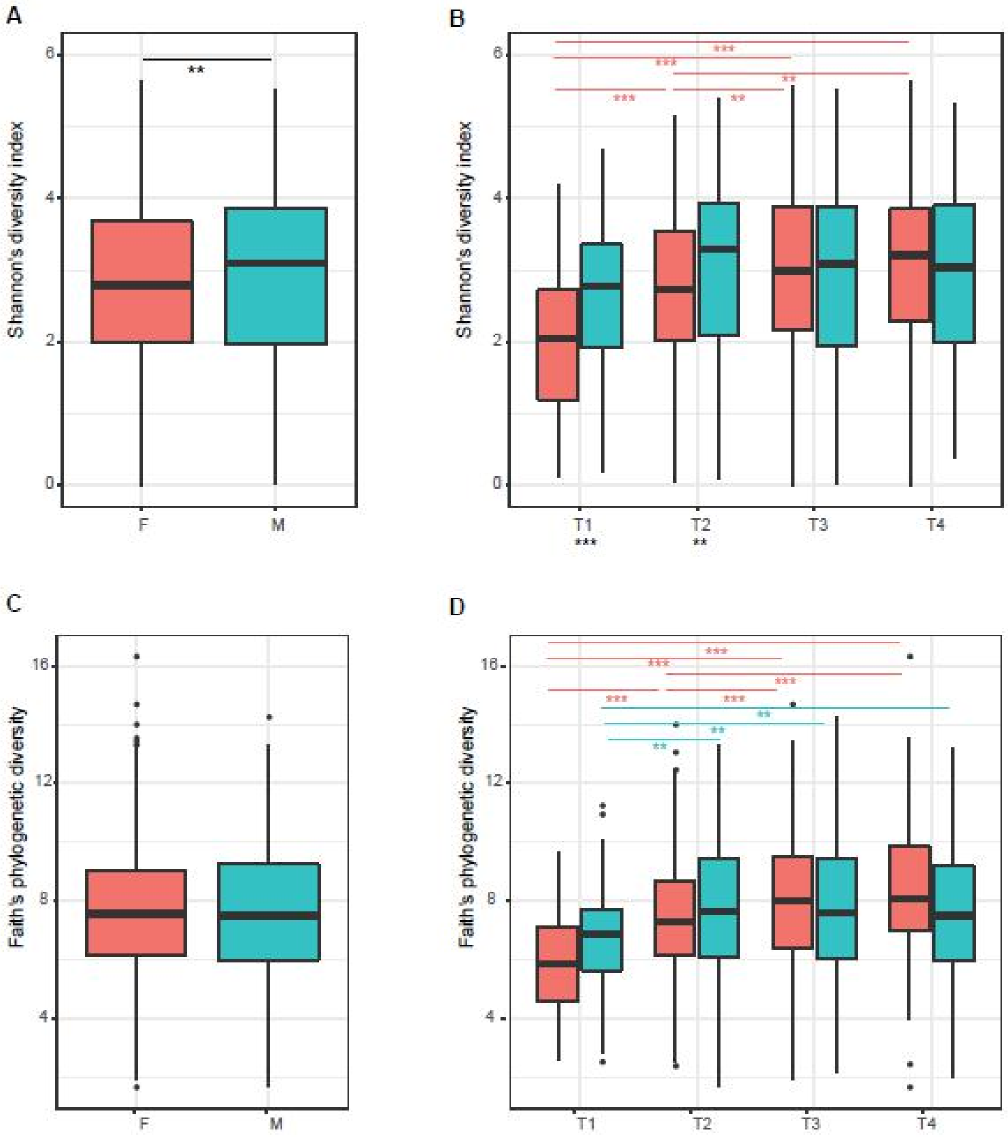

Figure S7. Comparison of alpha diversity index between genders (A, C) and age stages (B, D). Two estimators of the alpha diversity, including Shannon's diversity index (A-B) and Pielou's evenness (C-D), are shown. The asterisks indicate the significant level: **, p-value < 0.05 (wilcox test); ***, p-value < 0.01.

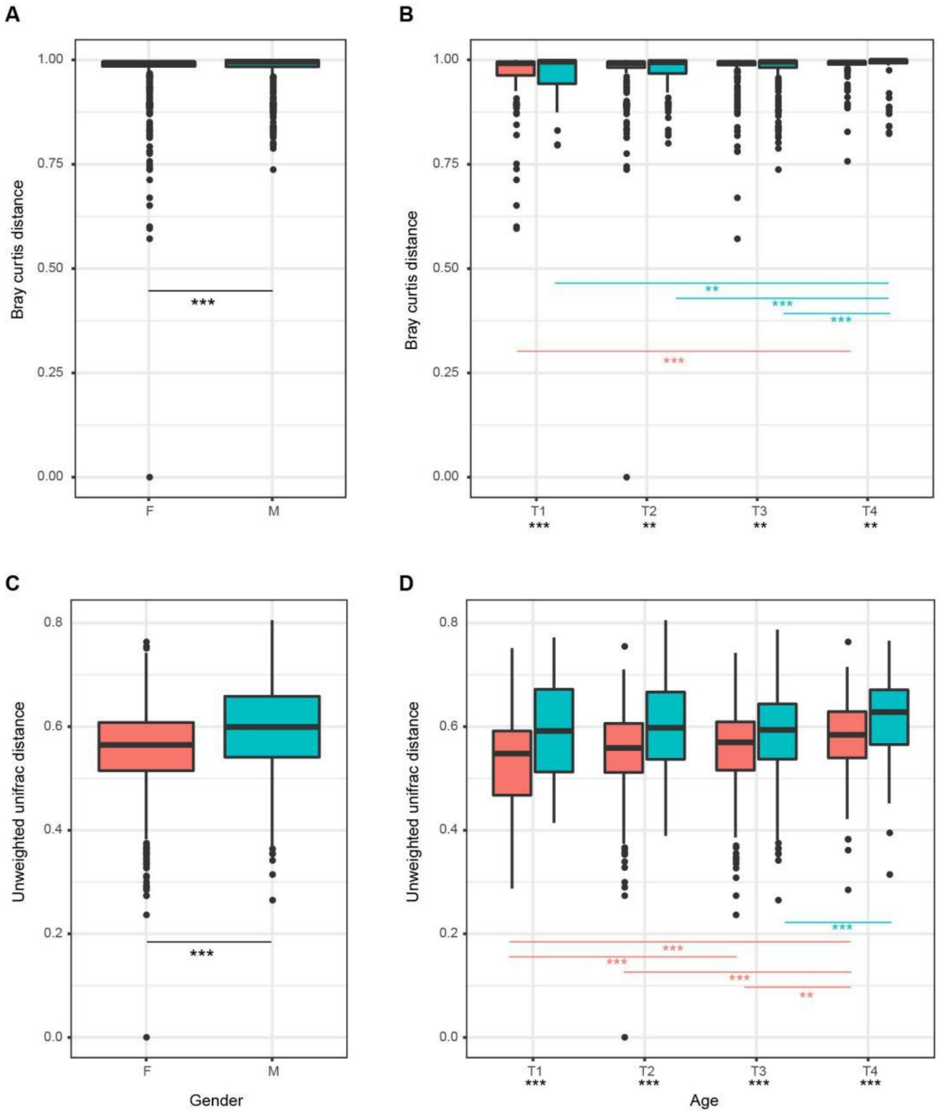

Figure S8. Comparison of beta diversity index between genders (A, C) and age stages (B, D). Two estimators of the beta diversity, including bray curtis distance (A-B) and unweighted unifrac distance (C-D), are shown. The asterisks indicate the significant level: **, p-value < 0.05 (wilcox test); ***, p-value < 0.01.

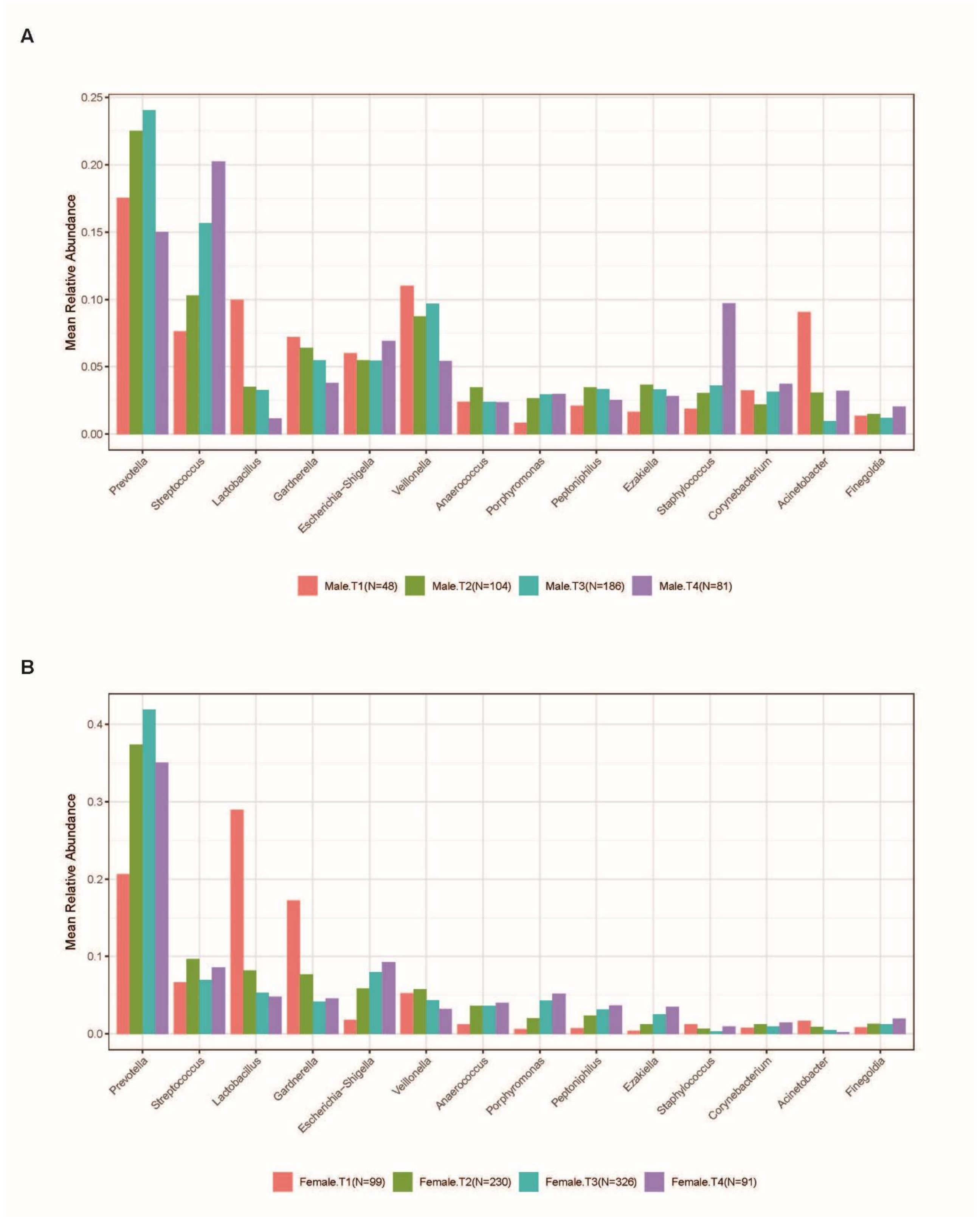

Figure S9. MRA comparison of 14 most dominant genera between age stages for males (A) and females (B) respectively.

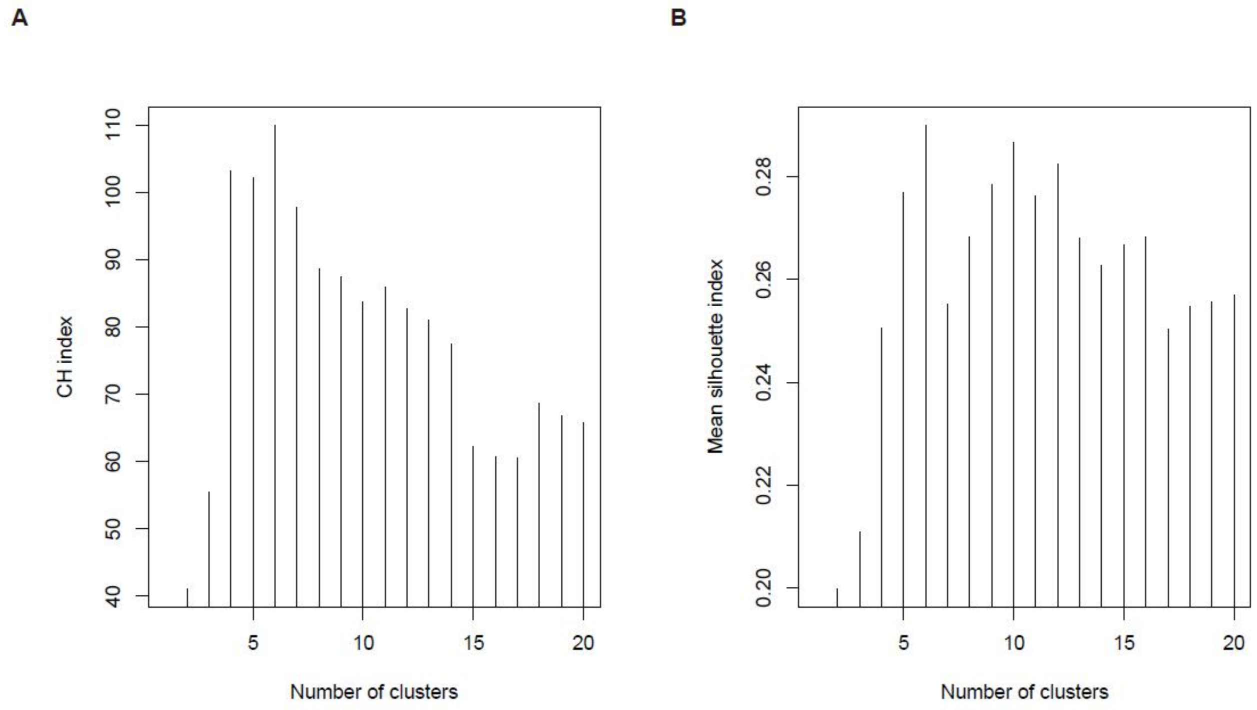

Figure S10. Calinski-Harabasz (CH) index (A) and mean silhouette width (B) analyses reveal the optimal number of urotypes in male samples.

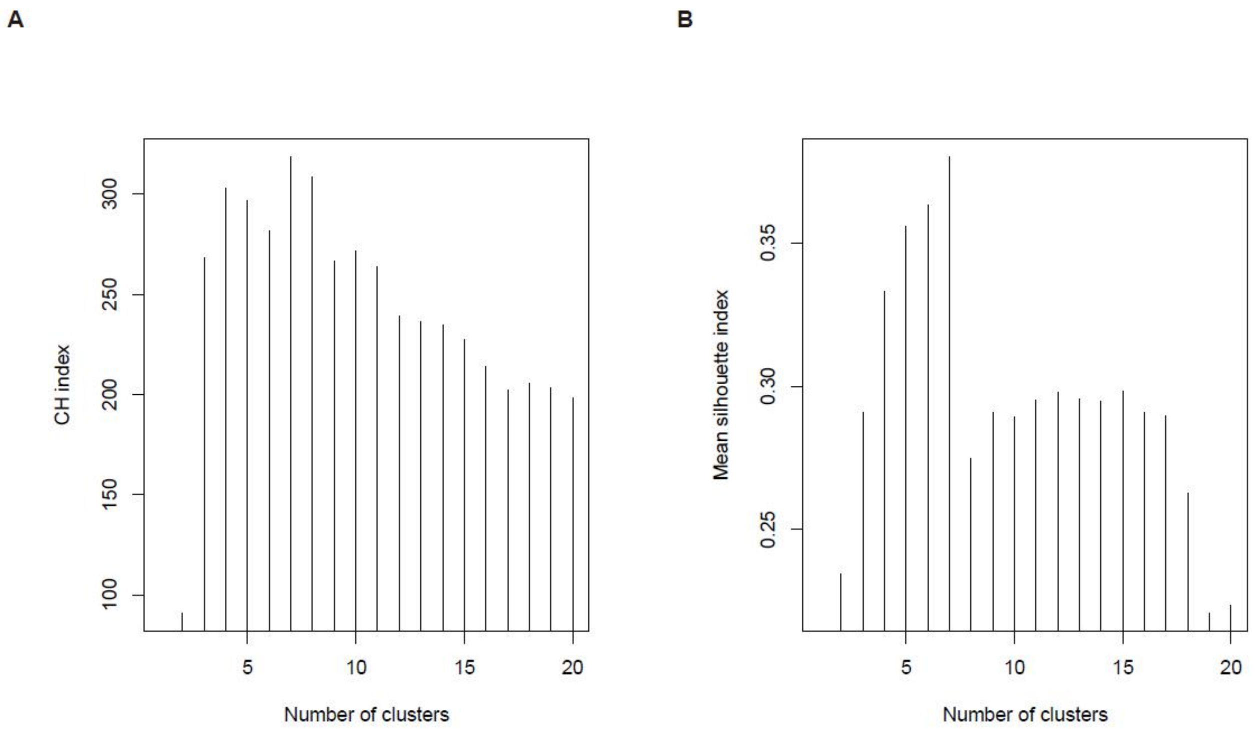

Figure S11. Calinski-Harabasz (CH) index (A) and mean silhouette width (B) analyses reveal the optimal number of urotypes in female samples.

Urotype	Prevotella	Streptococcus	Lactobacillus	Gardnerella	Escherichia-Shigella	Veillonella	Anaerococcus	Porphyromonas	Peptoniphilus	Ezakiella	Staphylococcus	Corynebacterium	Acinetobacter	Finegoldia	Sneathia	Dialister	Pseudomonas	Sphingomonas	Enterococcus	Atopobium
FUT1	100.00	76.20	23.51	37.68	41.93	50.14	93.48	91.22	96.03	83.00	46.74	72.24	35.13	82.44	22.66	98.87	7.08	14.16	8.78	42.21
FUT2	92.59	61.11	29.63	37.04	100.00	42.59	61.11	53.70	57.41	46.30	24.07	42.59	20.37	38.89	11.11	53.70	11.11	3.70	7.41	18.52
FUT3	96.15	67.31	44.23	98.08	36.54	34.62	75.00	55.77	71.15	50.00	42.31	53.85	23.08	71.15	23.08	80.77	11.54	13.46	7.69	55.77
FUT4	94.94	55.70	100.00	59.49	31.65	44.30	65.82	49.37	65.82	44.30	45.57	58.23	29.11	70.89	11.39	58.23	5.06	10.13	15.19	27.85
FUT5_1	100.00	75.44	10.53	29.82	49.12	36.84	100.00	100.00	100.00	98.25	49.12	85.96	36.84	94.74	21.05	100.00	10.53	8.77	7.02	45.61
FUT5_2	98.53	77.94	23.53	25.00	60.29	50.00	91.18	79.41	89.71	70.59	66.18	86.76	60.29	83.82	11.76	83.82	32.35	29.41	22.06	23.53
FUT6	100.00	100.00	32.65	32.65	55.10	57.14	95.92	71.43	95.92	69.39	85.71	87.76	51.02	97.96	10.20	89.80	14.29	24.49	24.49	18.37
FUT7	97.06	91.18	38.24	73.53	38.24	100.00	94.12	85.29	88.24	76.47	41.18	61.76	29.41	85.29	41.18	82.35	11.76	8.82	11.76	52.94
MUT1	99.29	67.38	25.53	45.39	30.50	36.88	95.74	96.45	97.87	87.23	53.19	73.76	38.30	80.14	29.79	86.52	15.60	18.44	11.35	40.43
MUT2	72.73	77.27	22.73	22.73	100.00	36.36	59.09	31.82	54.55	18.18	72.73	72.73	63.64	54.55	13.64	18.18	40.91	18.18	9.09	4.55
MUT3	82.72	100.00	18.52	22.22	40.74	35.80	86.42	59.26	83.95	41.98	90.12	93.83	67.90	82.72	11.11	39.51	25.93	43.21	7.41	8.64
MUT4	94.55	72.73	52.73	87.27	27.27	100.00	70.91	43.64	65.45	36.36	49.09	50.91	34.55	54.55	60.00	52.73	9.09	14.55	3.64	65.45
MUT5_1	71.43	52.38	100.00	76.19	52.38	38.10	61.90	28.57	57.14	33.33	76.19	90.48	80.95	66.67	19.05	42.86	9.52	33.33	4.76	38.10
MUT5_2	94.12	70.59	35.29	100.00	35.29	29.41	82.35	76.47	88.24	70.59	58.82	100.00	35.29	88.24	35.29	58.82	17.65	17.65	11.76	47.06
MUT6_1	71.43	42.86	42.86	14.29	92.86	14.29	64.29	42.86	50.00	42.86	78.57	78.57	100.00	42.86	0.00	14.29	78.57	21.43	28.57	7.14
MUT6_2	63.64	18.18	54.55	18.18	27.27	27.27	100.00	63.64	72.73	45.45	100.00	100.00	72.73	90.91	9.09	9.09	9.09	27.27	9.09	9.09
MUT6_3	85.71	71.43	21.43	21.43	42.86	42.86	100.00	57.14	85.71	50.00	100.00	100.00	78.57	92.86	0.00	14.29	50.00	28.57	14.29	14.29
MUT6_4	90.91	63.64	54.55	54.55	63.64	45.45	90.91	36.36	72.73	63.64	81.82	100.00	81.82	90.91	9.09	36.36	54.55	100.00	36.36	18.18
MUT6_Other	71.88	71.88	21.88	6.25	53.12	37.50	62.50	34.38	50.00	25.00	71.88	71.88	59.38	53.12	3.12	15.62	43.75	50.00	25.00	3.12

Figure S12. Prevalence of dominant genera in each urotype.

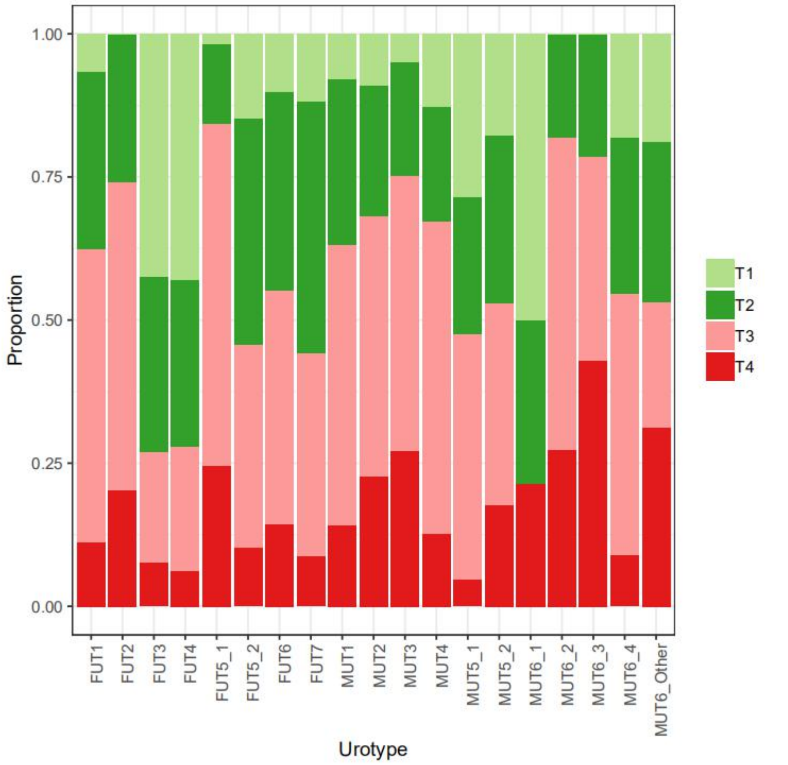

Figure S13. Proportion of samples at different age periods in each urotype.

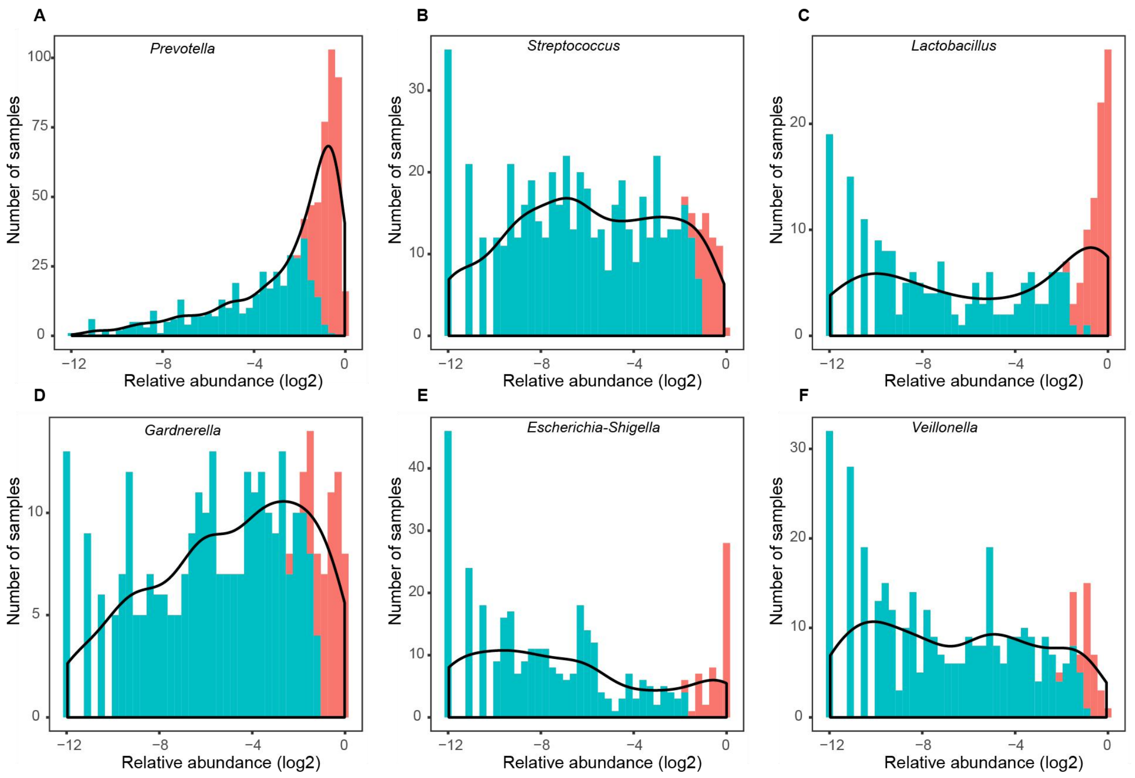

Figure S14. Histogram show the relative abundance distribution of the dominant genera in all male individuals. For each genus, the individuals belong to the corresponding urotype are shown by red bars, and the remains are shown by cyan bars.

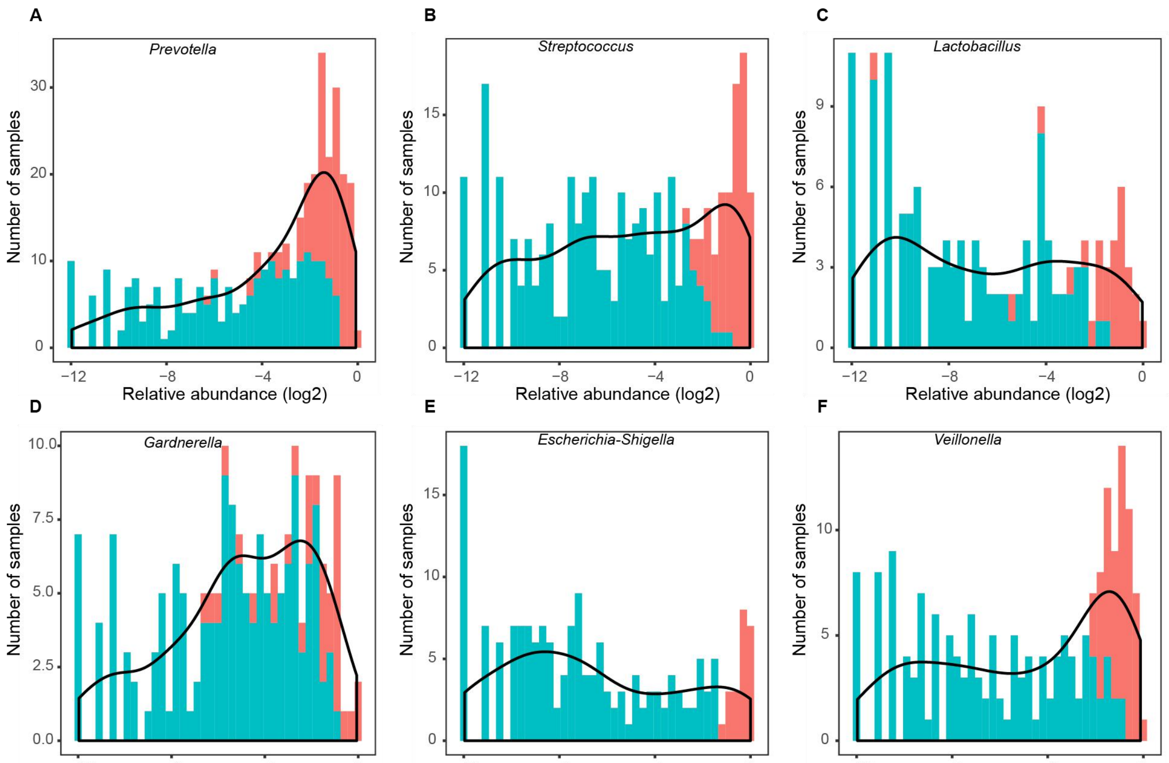

Figure S15. Histogram show the relative abundance distribution of the dominant genera in all female individuals. For each genus, the individuals belong to the corresponding urotype are shown by red bars, and the

remains are shown by cyan bars.

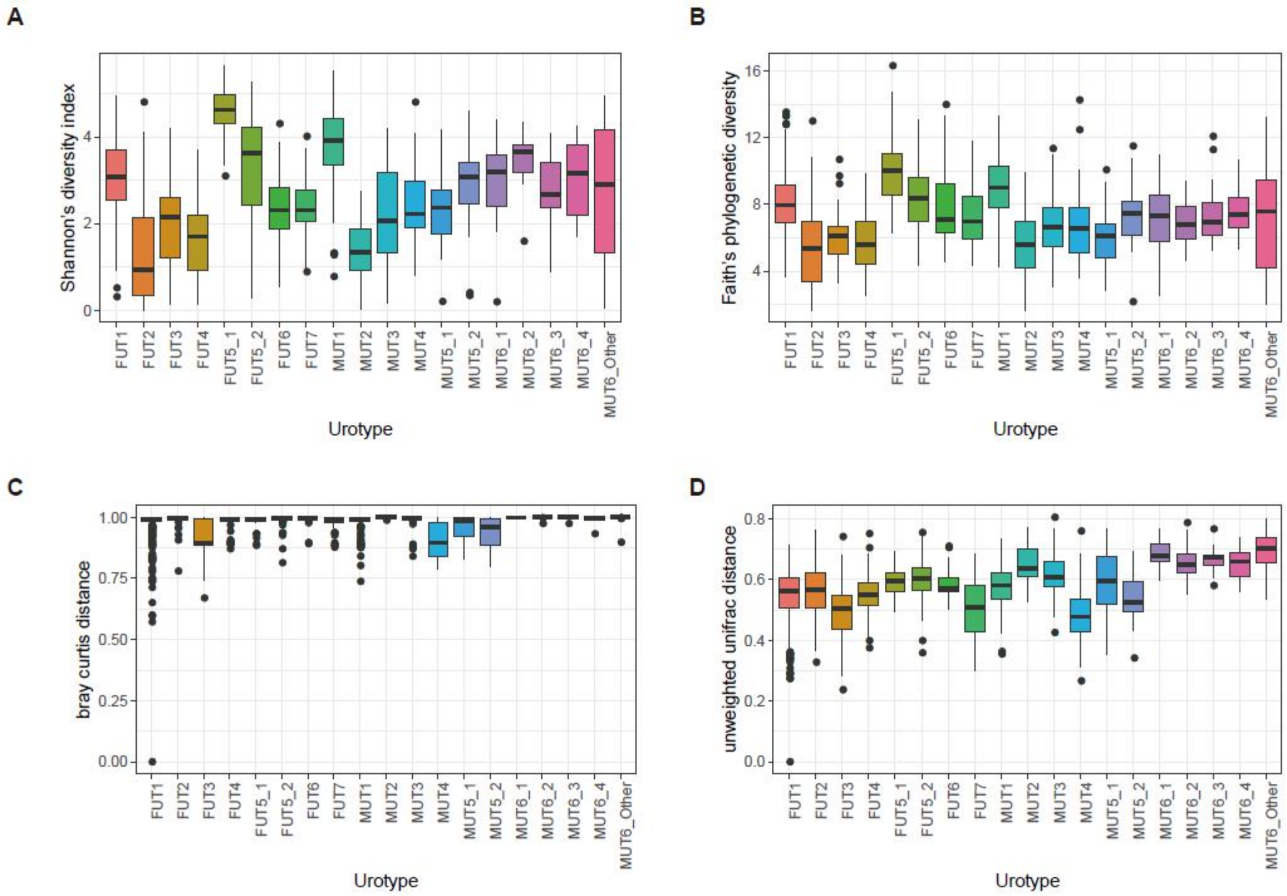

Figure S16. Comparison of alpha and beta diversity index between different urotypes.

Two estimators of the alpha diversity, including Shannon’s diversity index (A) and Faith's phylogenetic diversity (B), as well as two estimators of the beta diversity, including bray curtis distance (C) and unweighted unifrac distance (D), are shown

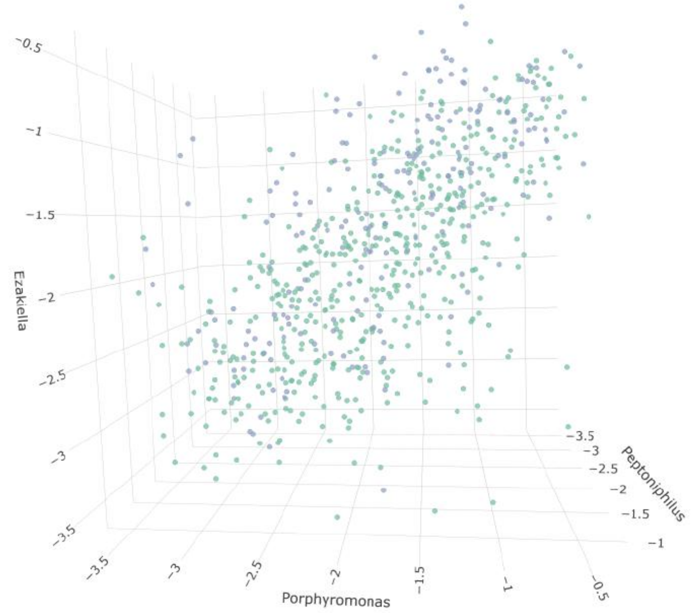

Figure S17. Co-abundance of *Peptoniphilus*, *Ezakiella* and *Porphyromonas.* Log10-scaled Relative abundance of each genus of each sample was plotted.

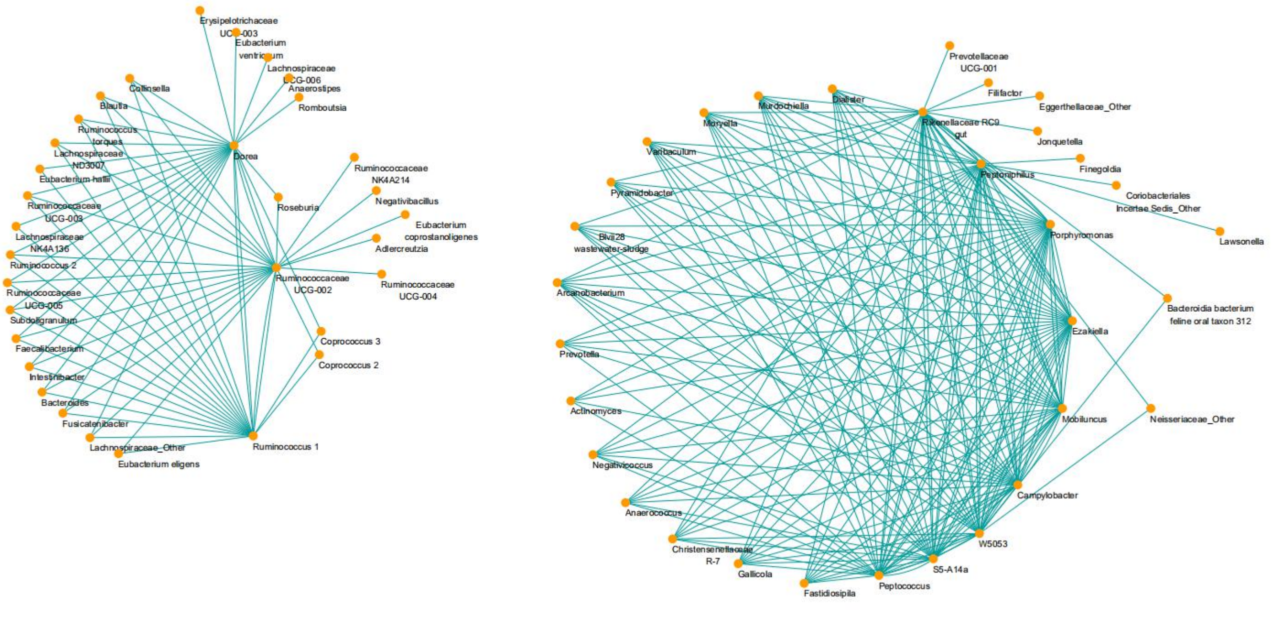

Figure S18. Network of major co-occurring genera based on spearman correlation analysis in all samples. Lines connected two genera with Spearman |rho| > 0.35, and only the keystone genera harboring at least 20 significant co-occured genera were plotted with their corresponding correlatives.

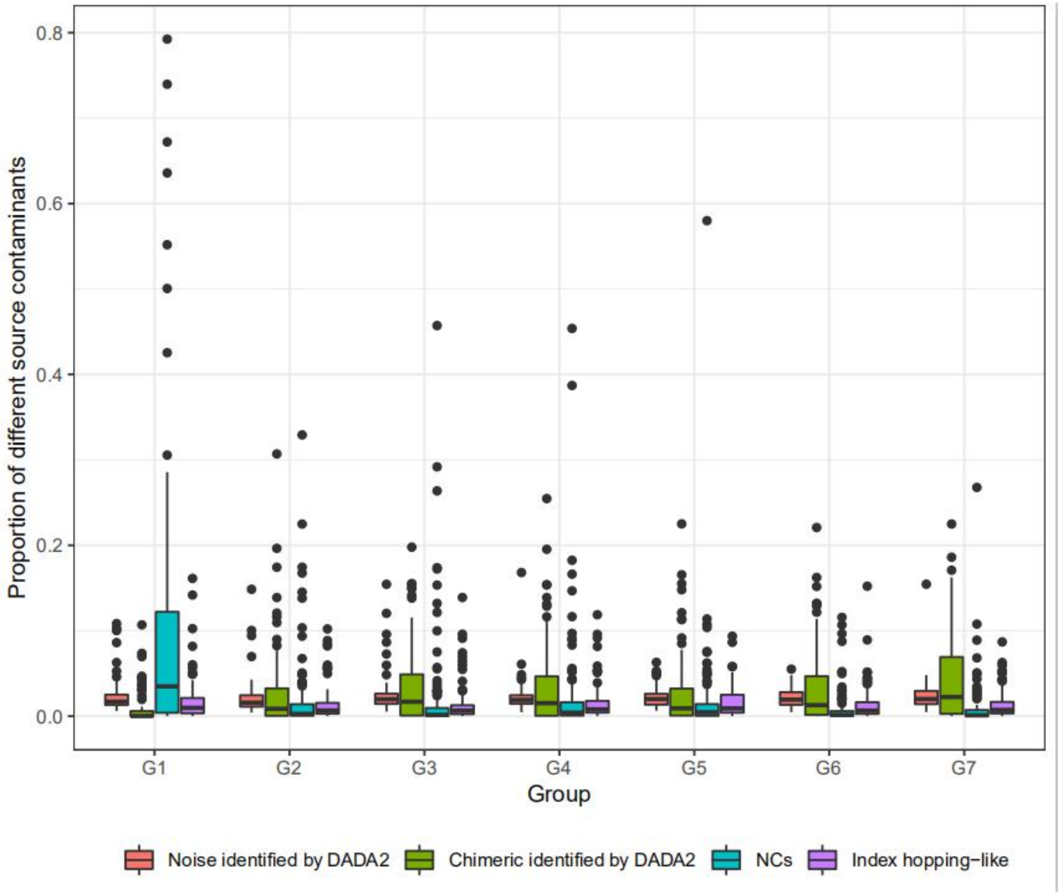

Figure S19. Proportion of reads affected by different sources of potential contaminants.

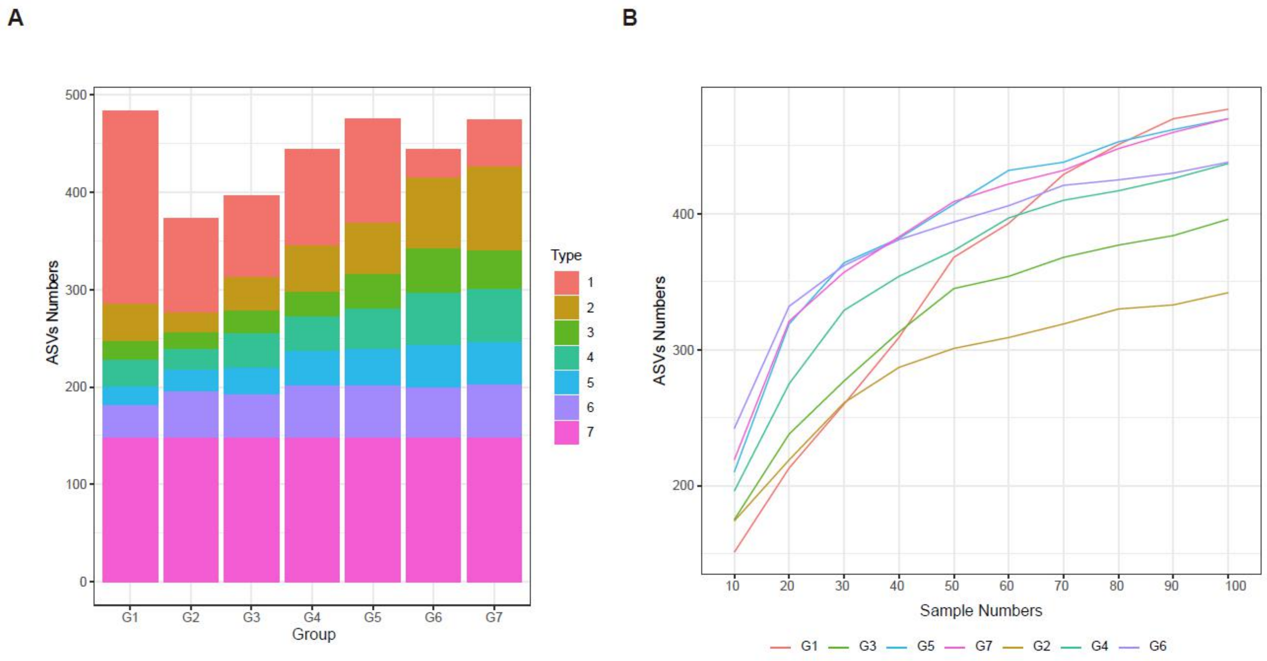

Figure S20. Impact of sequencing depth and sample size to identified ASVs numbers. According to sequencing depth, samples were classified into 7 groups, G1-7. 110 samples in each group were extracted, and ASVs were identified after removing potential contaminants. The presence of ASVs was compared between groups (A). ASVs in certain depth group were divided into 7 types according to the reoccurrence counts with

other groups. ASVs numbers identified from different numbers of samples were plotted in each depth group (B).

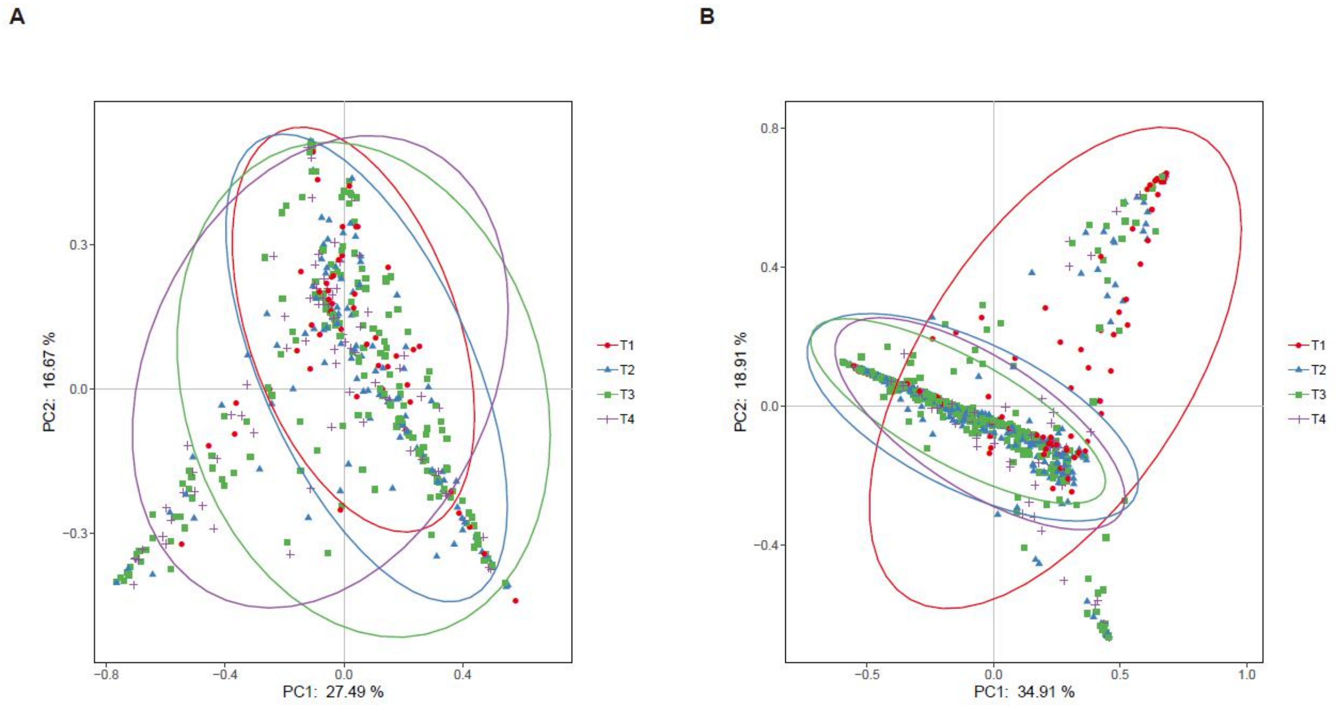

Figure S21. Principal component analysis (PCA) of dissolved genitourinary microbiome relative abundance composition for male (A) and female (B) samples respectively. Samples were marked with different colours according to their age stages.

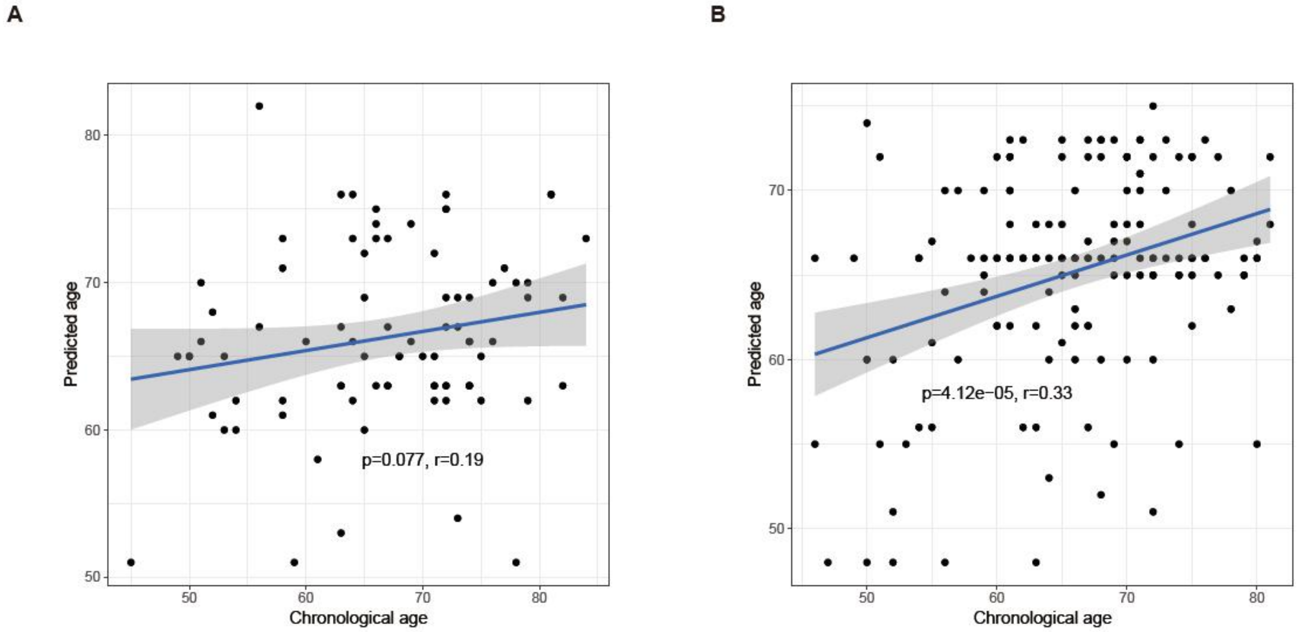

Figure S22. Correlation of chronological and random Forest-predicted age for male (A) and female (B) samples respectively. Correlation coefficient and p value were calcluated via *cor* function in R.
